# CRISPR/Cas13d targeting suppresses repeat-associated non-AUG translation of *C9orf72* hexanucleotide repeat RNA

**DOI:** 10.1172/JCI179016

**Published:** 2024-09-17

**Authors:** Honghe Liu, Xiao-Feng Zhao, Yu-Ning Lu, Lindsey R. Hayes, Jiou Wang

**Affiliations:** 1 Department of Biochemistry and Molecular Biology, Bloomberg School of Public Health, and; 2 Department of Neuroscience, School of Medicine, Johns Hopkins University, Baltimore, Maryland, USA.; 3 Brain Science Institute and Department of Neurology, Johns Hopkins University School of Medicine, Baltimore, Maryland, USA.

**Keywords:** Genetics, Neuroscience, Gene therapy, Molecular biology, Neurodegeneration

## Abstract

A hexanucleotide GGGGCC repeat expansion in the non-coding region of the *C9orf72* gene is the most common genetic mutation identified in patients with amyotrophic lateral sclerosis (ALS) and frontotemporal dementia (FTD). The resulting repeat RNA and dipeptide repeat proteins from non-conventional repeat translation have been recognized as important markers associated with the diseases. CRISPR/Cas13d, a powerful RNA-targeting tool, has faced challenges in effectively targeting RNA with stable secondary structures. Here we report that CRISPR/Cas13d can be optimized to specifically target GGGGCC repeat RNA. Our results demonstrate that the CRISPR/Cas13d system can be harnessed to significantly diminish the translation of poly-dipeptides originating from the GGGGCC repeat RNA. This efficacy has been validated in various cell types, including induced pluripotent stem cells and differentiated motor neurons originating from C9orf72-ALS patients, as well as in *C9orf72* repeat transgenic mice. These findings demonstrate the application of CRISPR/Cas13d in targeting RNA with intricate higher-order structures and suggest a potential therapeutic approach for ALS and FTD.

## Abstract 2

CRISPR-Cas13d targeting of GGGGCC repeat RNA reduces toxic dipeptide proteins in patient cells and mice, suggesting a potential therapy for ALS/FTD and related diseases.

## Graphical abstract

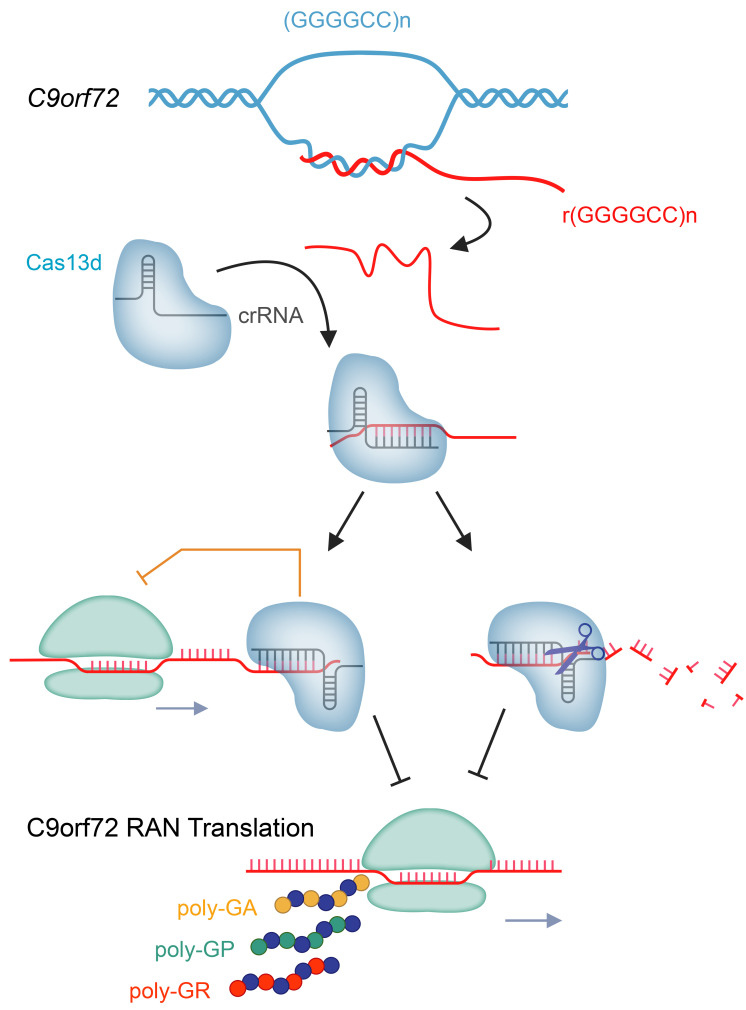

## Introduction

Amyotrophic lateral sclerosis (ALS) is a progressive neurodegenerative disease that impacts both the upper and lower motor neurons. *C9orf72* hexanucleotide repeat GGGGCC expansion is the most frequent genetic cause of ALS and also the related disease frontotemporal dementia (FTD) (1, 2). Typically, the majority of individuals have two GGGGCC repeat units, but in affected individuals, this expands to hundreds or thousands (3). Regarding the GGGGCC repeat expansion, several disease mechanisms have been proposed, including loss of function of the C9orf72 protein, gain of function via the expanded repeat at the DNA or RNA level, and the generation of dipeptide repeat proteins (DPRs) through repeat-associated non-AUG (RAN) translation (4, 5). Accordingly, therapeutic strategies including antisense oligonucleotides (ASOs), RNA-targeting Cas9 (RCas9), small interfering RNAs (siRNAs), artificial microRNAs (miRNAs), and DPR antibodies have been developed to target either repeat-associated RNAs or DPRs (6–12). However, these existing strategies have their limitations. For example, ASOs require lifelong and routine administration. RCas9 shows reduced efficiency in targeting GGGGCC repeat RNAs (7). Studies have shown that siRNAs and miRNAs tend to be inefficient at RNA target sites with strong secondary structures (13, 14), and the siRNAs and miRNAs reported for targeting C9orf72-ALS were not designed to target GGGGCC repeat RNA sequences (8, 9). Furthermore, therapeutic antibodies aimed at intracellular targets such as DPRs face challenges related to the accessibility and complexity of the targets. Thus, new strategies are needed to target the repeat RNA or its translational products more comprehensively.

CRISPR/Cas13 belongs to type VI CRISPR/Cas systems and has been identified as RNA-targeting Cas proteins guided by a single CRISPR RNA (crRNA) (15). To date, a variety of subtypes of Cas13 proteins, including Cas13a, Cas13b, Cas13c, and Cas13d, have been identified and applied to gene knockdown (16), RNA imaging and tracking (17), viral RNA detection (18), and site-directed RNA editing (19). Particularly, Cas13b and Cas13d have robust RNA cleavage abilities and minimal off-target activities in cells compared with other Cas13s (16, 19). However, previous studies also showed that both Cas13b and Cas13d have sequence preference and not all RNAs could be efficiently targeted, especially RNAs adopting strong secondary structure (20, 21). *C9orf72* GGGGCC repeat RNAs are highly GC-rich RNAs that could form stable G-quadruplex structures, which have been validated by us and others (22–25), rendering targeting of *C9orf72* repeat RNA by Cas13b and Cas13d potentially challenging. Additionally, the impact of CRISPR/Cas13 on targeting of RNA-based translation has not been explored.

In this study, we develop a strategy based on the CRISPR/Cas13d (CasRx) system to directly target GGGGCC repeat RNAs, achieving significant reduction of toxic DPRs in ALS patient cells and a mouse model, even under conditions of limited degradation of GGGGCC repeat RNAs. Benefiting from the small size, good efficiency, and specificity of Cas13d, our strategy provides a new therapeutic option for ALS and FTD. Furthermore, our investigation provides fresh insights into the ability of the CRISPR/Cas13 system to target RNAs with higher-order structures at the translation level.

## Results

### Development of CRISPR/Cas13 systems targeting GGGGCC repeat RNAs.

**Figure 1:**
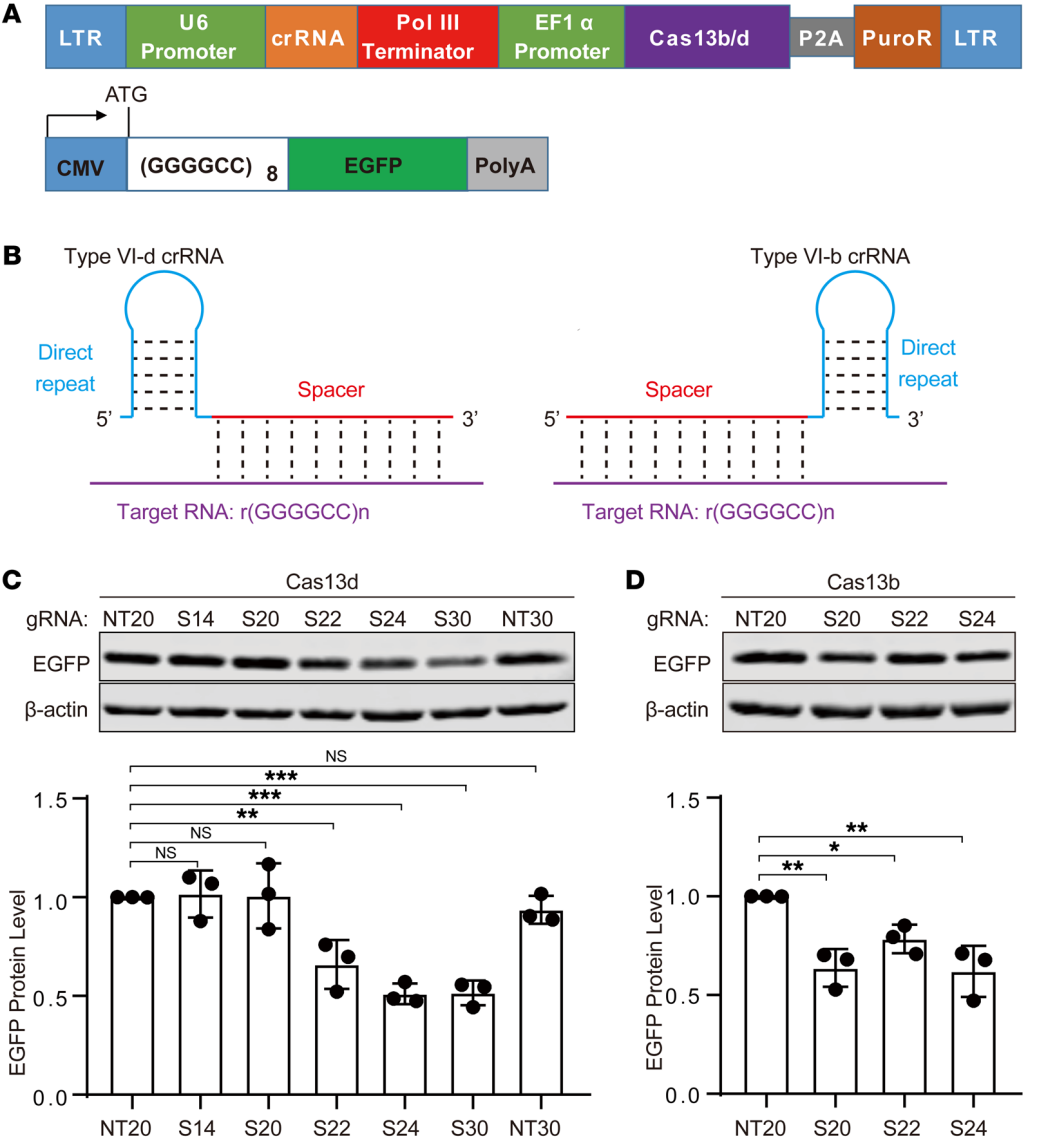
The one-vector CRISPR/Cas13d system and the test for its knockdown efficiency. (**A**) Schematic of the one-vector CRISPR/Cas13b/d system construct (top) and the EGFP reporter construct (bottom). (**B**) Schematic of type VI-d (left) and VI-b (right) crRNA structures with the target RNA. The crRNAs carry a direct repeat sequence (blue) to facilitate the binding with their corresponding Cas13 enzyme, and a spacer sequence (red) specific for the target RNA, r(GGGGCC)*n* (purple). (**C** and **D**) The knockdown efficiency test in HEK293 cells via cotransfection of the CRISPR/Cas13d vector (**C**), or the CRISPR/Cas13b vector (**D**), and the reporter construct. Immunoblotting of EGFP showed the knockdown efficiency for different guide RNAs (gRNAs). Data are presented as means ± SD of 3 independent experiments and were analyzed with ordinary 1-way ANOVA with Dunnett’s multiple-comparison test. *P < 0.05, **P < 0.01, ***P < 0.001.

To employ the CRISPR/Cas13 system to target GGGGCC repeat RNAs, we began by designing a one-vector system, using a lentiviral vector that incorporated both Cas13d or Cas13b protein and a corresponding crRNA. Additionally, a reporter construct containing 8× GGGGCC repeats followed by the EGFP gene was created to monitor the efficiency of the repeat-dependent knockdown by the CRISPR/Cas13 system via the measurement of EGFP translation levels. EGFP translation from a transcript containing the GGGGCC repeats, initiated by the ATG start codon, would be interrupted upon degradation of the repeat-containing RNA (Figure 1A).

Given the ability of GGGGCC repeat RNA to form highly stable secondary structures such as G-quadruplexes or hairpins (24, 26), and considering that the secondary structures of crRNA can impact targeting efficiency (21), we sought to optimize the crRNA design. Our approach involved altering the length and the beginning nucleotide of the spacer region while minimizing any disruption to the stem loop structure of the direct repeat region of crRNA and avoiding secondary structures within the spacer RNA (Figure 1B). For the Cas13d system, we designed a set of 7 crRNAs, including 2 non-targeting control crRNAs (NT20 and NT30) and 5 crRNAs (S14, S20, S22, S24, S30) targeting GGGGCC repeat RNAs with varying lengths. Similarly, for the Cas13b system, we designed 4 crRNAs, consisting of 1 non-targeting control crRNA (NT20) and 3 crRNAs (S20, S22, S24) targeting GGGGCC repeat RNAs with different lengths. To evaluate the knockdown efficiency, we transfected HEK293 cells with the designed CRISPR/Cas13 constructs, along with the EGFP reporter construct. Subsequently, we measured the EGFP protein level 48 hours after transfection. Our hypothesis was that the CRISPR/Cas13 construct would cleave the (GGGGCC)8 RNA, resulting in the inhibition of EGFP mRNA translation due to the loss of the start codon ATG in the transcripts. The results for the CRISPR/Cas13d system demonstrate that crRNAs S22, S24, and S30 exhibited significantly reduced EGFP levels, while S14 and S20 showed minimal changes (Figure 1C). Among these crRNAs, S24 and S30 displayed the highest efficiency, with approximately 50% knockdown compared with the control crRNA. For the CRISPR/Cas13b system, crRNAs S20, S22, and S24 all demonstrated substantial knockdown efficiency compared with the non-targeting control crRNA (NT20) (Figure 1D). However, the CRISPR/Cas13b system showed lower efficiency compared with the CRISPR/Cas13d system with the crRNAs S24 and S30. Additionally, we quantified the EGFP RNA level, which yielded results consistent with the observed trends for EGFP protein levels (Supplemental Figure 1, A and B; supplemental material available online with this article; https://doi.org/10.1172/JCI179016DS1), thus confirming the ability of the CRISPR/Cas13 system to degrade GGGGCC repeat RNAs. In summary, we developed CRISPR/Cas13 systems for targeting GGGGCC repeat RNAs, with crRNA S24 and S30 in the CRISPR/Cas13d system demonstrating the most robust knockdown efficiency.

### CRISPR/Cas13d suppresses C9orf72 RAN translation in dual-luciferase–based reporters.

**Figure 2:**
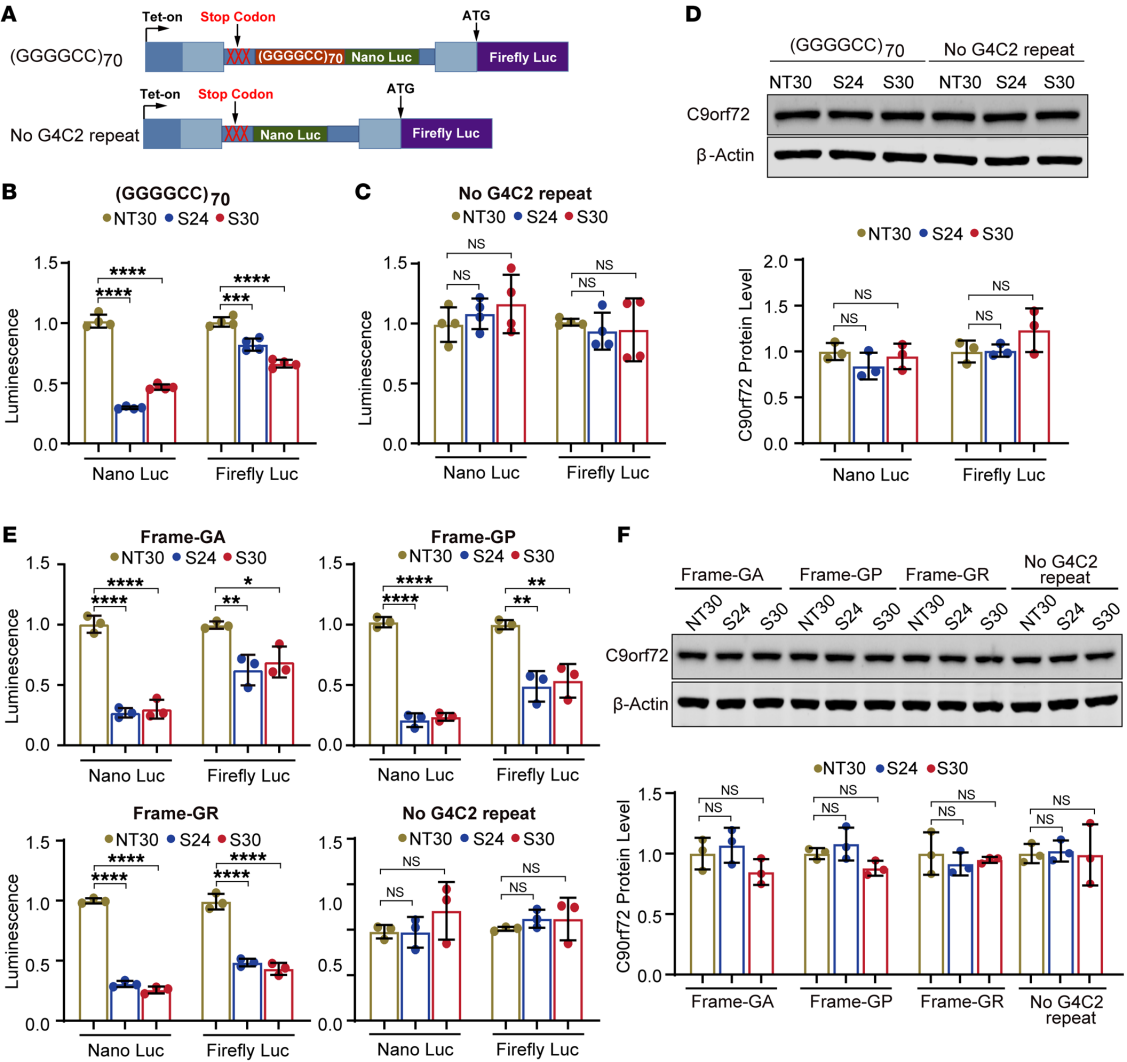
Guided Cas13d specifically decreased translation associated with GGGGCC repeat–containing transcripts. (**A**) Schematic of the inducible luciferase-based C9orf72 RAN translation reporter system in HeLa Flp-In cells. (**B**) The reporter cells stably expressing Cas13d and gRNA S24 and S30 showed lower signals of NanoLuc and firefly luciferase signal from the (GGGGCC)70-containing reporter transcripts. The significant reduction of the NanoLuc luciferase signal relative to the firefly luciferase signal demonstrates that the repeat-associated RAN translation is inhibited by Cas13d-mediated S24 or S30 treatment. (**C**) The control cells harboring the reporter without the G4C2 repeat showed no effect of the Cas13d gRNAs on the translation of the reporters. (**D**) Immunoblot analysis of C9orf72 protein showed that the C9orf72 protein level was unaffected in HeLa RAN translation reporter cell lines stably expressing Cas13d-NT30, Cas13d-S24, and Cs13d-S30. (**E**) Transient cotransfection of CRISPR/Cas13d constructs with either the GA-frame, GP-frame, or GR-frame or the No-G4C2-repeat control construct in HEK293 cells showed that Cas13d-S24 and Cas13d-S30 significantly reduced both NanoLuc and firefly luciferase signals from the GA-, GP-, and GR-frame but not the negative No-G4C2-repeat control reporter compared with the non-targeting control Cas13d-NT30. (**F**) Immunoblot analysis of C9orf72 protein showed that the C9orf72 protein level was unaffected in HEK293 cells cotransfected with CRISPR/Cas13d and either the GA-frame, GP-frame, or GR-frame or the No-G4C2-repeat control construct. Data are presented as means ± SD of 3 or 4 biological replicates as indicated by the number of dots in each graph, and were analyzed with ordinary 1-way ANOVA with Dunnett’s multiple-comparison test. *P < 0.05, **P < 0.01, ***P < 0.001, ****P < 0.0001.

After confirming the effective targeting capability of S24 and S30, we applied the CRISPR/Cas13d systems to a previously established reporter cell line system that used dual-luciferase–based reporter cassettes designed for monitoring C9orf72 RAN translation (27). In the reporter cassettes, a (GGGGCC)70 repeat was inserted upstream of coding sequence of the NanoLuc luciferase (Nluc) without ATG start codon in the intron region in the poly-GP (Gly-Pro) frame to monitor the translation products resulting from the repeat-initiated RAN translation. The firefly luciferase (Fluc) with an ATG start codon was cloned into the same construct in the exon region as an internal control for canonical translation, allowing for the measurement of RAN translation based on relative ratios of Nluc to Fluc activities. The reporter cassette, with or without the (GGGGCC)70 repeat, was integrated into the unique Flp-In site in the stable reporter HeLa cell lines (Figure 2A), and it has been verified that there is no difference in the splicing efficiency of pre-mRNA of this reporter cassette with and without (GGGGCC)70 (27).

Next, we transduced the reporter HeLa cell lines with lentiviruses expressing the CRISPR/Cas13d system with the NT30, S24, or S30 crRNA, and generated reporter cell lines stably expressing Cas13d protein and corresponding crRNAs. We induced the RAN translation reporter expression using doxycycline and measured the Nluc and Fluc activity in the reporter cell lines with or without (GGGGCC)70. As expected, both S24 and S30 crRNAs significantly deceased the Nluc signal in the (GGGGCC)70 reporter cell line (Figure 2B), indicating that both S24- and S30-guided Cas13d could specifically recognize the GGGGCC repeat RNA and suppress its translation. Notably, it has been reported that the unspliced transcript of this reporter cassette was also detected in the cytoplasm (27), enabling the unspliced transcript containing the (GGGGCC)70 to be templated for the translation of Fluc, thereby subjecting both Nluc and Fluc RNAs on this unspliced transcript to the targeting of the S24- and S30-guided Cas13d. As expected, the Fluc signal was also decreased but with less efficiency than the Nluc signal, consistent with notion that the guided Cas13d recognized the unspliced transcript containing (GGGGCC)70, thus affecting the expression of both Nluc and Fluc. Importantly, the significant reduction of the Nluc signal relative to the Fluc signal demonstrates that the Cas13d system is more effective in suppressing the repeat-dependent RAN translation. Furthermore, the luciferase activity in reporter cell lines without (GGGGCC)70 was not affected by either S24 or S30 (Figure 2C), further confirming the specificity and efficiency of the CRISPR/Cas13d system in targeting the GGGGCC repeat RNA and suppressing RAN translation.

To determine whether the inhibition of RAN translation was achieved through degradation of GGGGCC repeat RNA or through translation blocking, we quantified the RNA levels of both the Nluc and Fluc reporters. The RNA levels of both the Nluc and Fluc reporters remained unaffected by S24- or S30-guided Cas13d compared with the non-targeting control NT30-guided Cas13d, in both the (GGGGCC)70 reporter and No-G4C2-repeat control cell lines (Supplemental Figure 1C). This result indicates that the CRISPR/Cas13d system does not influence the RNA levels of the reporter constructs, suggesting that RAN translation inhibition occurs via translation blocking rather than RNA degradation. Notably, the *C9orf72* gene generated the 3 transcripts through alternative start sites and splicing, and the GGGGCC repeat was transcribed in C9-V1 and C9-V3 but not in C9-V2 (Supplemental Figure 1D). To determine whether the CRISPR/Cas13d system affects transcripts from the wild-type (WT) *C9orf72* repeat allele carrying only two GGGGCC repeats, we quantified the 3 known transcripts of the *C9orf72* gene in the HeLa cells treated with the CRISPR/Cas13d system. None of the 3 transcripts of the *C9orf72* gene (C9-V1, C9-V2, and C9-V3) was affected (Supplemental Figure 1C), suggesting that the CRISPR/Cas13d system does not impact transcripts from the normal WT *C9orf72* repeat allele. Immunoblotting assays yielded consistent results, showing that the endogenous C9orf72 protein level was also unaffected (Figure 2D), indicating the selective targeting ability of the CRISPR/Cas13d system for expanded long GGGGCC repeats.

To determine whether the CRISPR/Cas13d system targeting GGGGCC repeat RNAs could affect other RAN translation products in addition to poly-GP validated above, we used 2 additional luciferase-based reporter constructs, harboring (GGGGCC)70 in poly-GA-frame and poly-GR-frame, each with 1 nucleotide shift compared with the poly-GP-frame (Figure 2A). Similar to the observations for poly-GP, the reporters in the poly-GA-frame and poly-GR-frame were observed to exhibit significantly higher Nluc activity, normalized to Fluc activity, when compared with the negative control construct lacking the GGGGCC repeat in HEK293 cells (Supplemental Figure 1E), confirming the RAN translation in the poly-GA-frame and poly-GR-frame. Next, we conducted transient cotransfection of these reporter constructs with the CRISPR/Cas13d construct in HEK293 cells and found that Cas13d-S24 and Cas13d-S30 significantly decreased the RAN translation at all 3 frames compared with the non-targeting control Cas13d-NT30 (Figure 2E), consistent with the results in the HeLa reporter cell lines (Figure 2, B and C). This result further confirms the effectiveness of the CRISPR/Cas13d system in different cell types. In addition, we also evaluated the levels of *C9orf72* RNA and protein in the transfected HEK293 cells and observed no significant changes, consistent with results in the HeLa reporter cell lines (Figure 2F and Supplemental Figure 1F).

### CRISPR/Cas13d suppresses RAN translation in cells derived from C9orf72-ALS patients.

**Figure 3:**
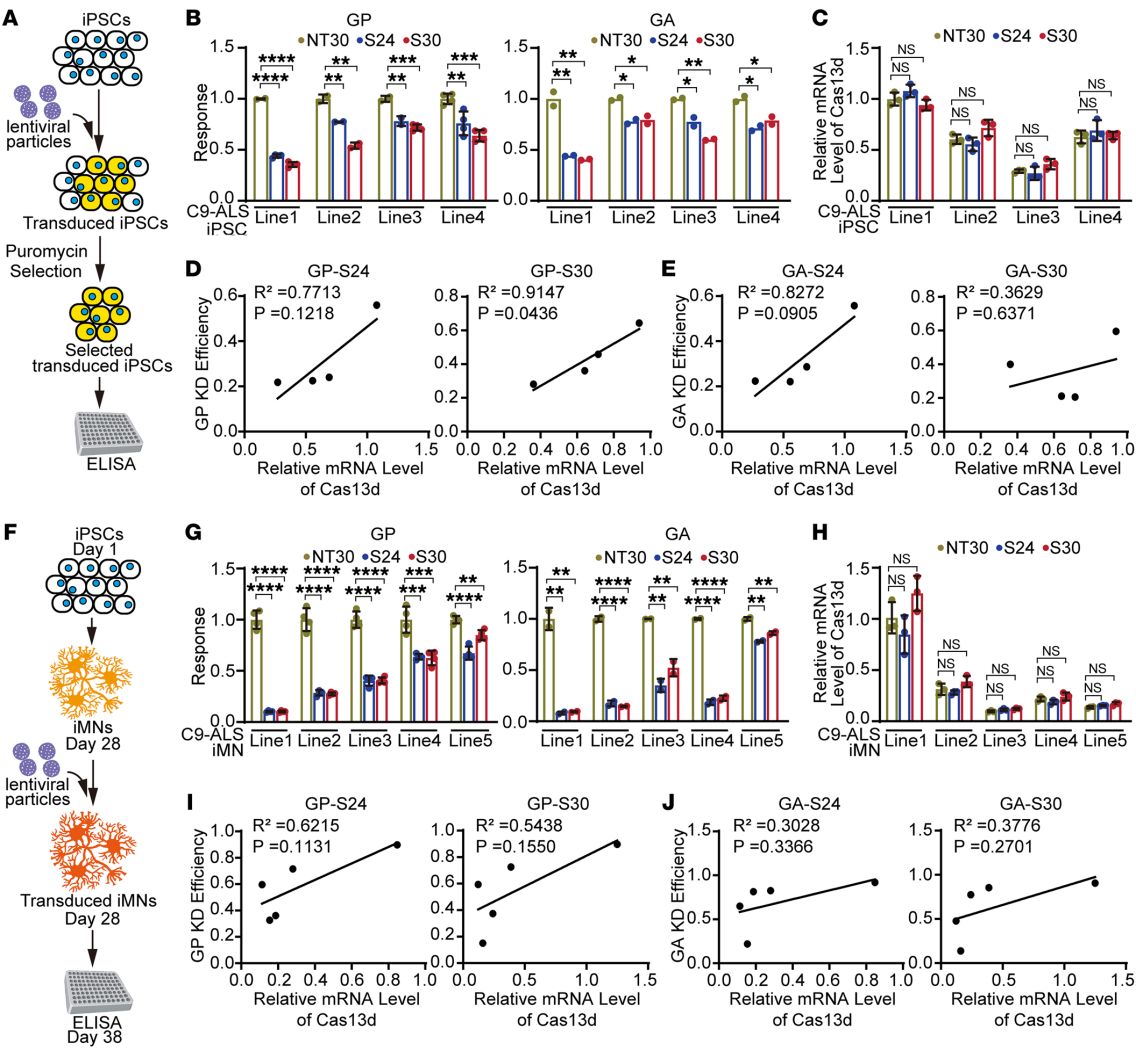
Guided Cas13d suppressed RAN translation in cells derived from patients carrying the C9orf72 hexanucleotide repeat expansion. (**A**) Schematic of RAN translation product detection in human iPSCs stably expressing Cas13d and gRNA via lentivirus transduction. (**B**) ELISA quantification in multiple C9-ALS patient iPSC cell lines showed significant reduction of poly-GP and poly-GA levels by Cas13d-S24 and CRISPR/S30 compared with the non-targeting control Cas13d-NT30. (**C**) Quantification of relative RNA levels of Cas13d in the C9-ALS patient iPSC cell lines showed variable Cas13d levels among lines, while in each line there were no significant differences among the S24, S30, and non-targeting NT30 groups. (**D** and **E**) Linear regression and correlation analyses showed a strong positive correlation between Cas13d expression level and poly-GP (**D**) and poly-GA (**E**) knockdown efficiency among C9-ALS patient iPSC lines. Pearson’s correlation coefficients and 2-tailed *P* value were computed. (**F**) Schematic of poly-GP and poly-GA detection in iMNs derived from human C9-ALS patient iPSCs. (**G**) ELISA quantification in iMN lines derived from multiple iPSC cell lines showed significant reduction in poly-GP and poly-GA levels by Cas13d-S24 and CRISPR/S30 compared with the non-targeting control Cas13d-NT30. (**H**) Quantification of relative RNA levels of Cas13d in the C9-ALS patient iMN lines showed variable Cas13d levels among lines, while in each line there were no significant differences among the S24, S30, and non-targeting NT30 groups. (**I** and **J**) Linear regression and correlation analyses showed a strong positive correlation between Cas13d expression level and poly-GP (**I**) and poly-GA (**J**) knockdown efficiency among C9-ALS patient iMN lines. Pearson’s correlation coefficients and 2-tailed *P* value were computed. Data are presented as means ± SD of 2–4 biological replicates as indicated by the number of dots in each graph, and were analyzed with ordinary 1-way ANOVA with Dunnett’s multiple-comparison test. *P < 0.05, **P < 0.01, ***P < 0.001, ****P < 0.0001.

Next, we used the CRISPR/Cas13d system to target endogenous GGGGCC repeat RNAs in cells obtained from patients with C9orf72-associated ALS. Multiple distinct lines of human induced pluripotent stem cells (iPSCs) were obtained from individuals with C9orf72-associated ALS, each containing the expanded GGGGCC repeat. These iPSC lines were treated with lentiviral particles carrying Cas13d and specific crRNAs (NT30, S24, or S30). After puromycin selection for 7 days, we obtained iPSCs that stably expressed Cas13d and the corresponding crRNAs. The resulting cells were collected and analyzed for the level of poly-GP and poly-GA as representative products of the repeat-dependent RAN translation, using an optimized sensitive ELISA detection assay (Figure 3A) (28). The results revealed significant reduction in both poly-GP and poly-GA levels in all patient cell lines when treated with Cas13d-S24 and Cas13d-S30, compared with NT30 (Figure 3B). The knockdown efficiency of GP and GA ranged from approximately 30% to 70% in different patient cell lines (Figure 3B), confirming the effectiveness of the CRISPR/Cas13d system in suppressing RAN translation in *C9orf72* repeat–associated ALS patient cells.

Given the varied RAN translation suppression efficiencies observed across different C9-ALS patient iPSC lines, we asked whether these differences were related to the expression levels of the transduced CRISPR/Cas13d system. We used quantitative reverse transcription PCR (RT-qPCR) to assess the expression level of Cas13d in each treated cell line and found variable Cas13d levels among lines. However, within each line, there were no significant differences in the average Cas13d levels among the S24, S30, and non-targeting NT30 groups (Figure 3C), confirming that the observed poly-GP and poly-GA knockdown was due to specific guide RNA (gRNA) targeting. We then performed linear regression and correlation analyses to examine the relationship between the Cas13d expression level and the efficiency of poly-GP and poly-GA knockdown across the iPSC lines. Importantly, we found a strong positive correlation between the average Cas13d expression level and the knockdown efficiency of both poly-GP and poly-GA across the C9-ALS iPSC lines (Figure 3, D and E). Notably, the correlation was particularly robust for Cas13d-S30 in the knockdown of poly-GP, with an *R*^2^ of 0.9147 and a *P* value of 0.0436 (Figure 3D). This positive dose-response relationship further substantiates the specificity of the designed crRNAs.

Since motor neurons are the primarily affected cells in ALS patients, we further assessed the CRISPR/Cas13d system in iPSC-derived motor neurons (iMNs) originating from C9orf72-associated ALS patients (Figure 3F). We differentiated multiple iPSC lines into mature iMNs using a previously established method (29). The resulting mature iMNs were validated by immunofluorescence staining of the mature motor neuron marker choline acetyltransferase (ChAT) (Supplemental Figure 2A). Subsequently, mature iMNs were treated with lentiviral particles expressing Cas13d and the corresponding crRNAs (NT30, S24, or S30) as described above. Ten days after the lentiviral treatment, iMNs were collected for poly-GP and poly-GA quantifications. All iMN lines exhibited a significant decrease in poly-GP and poly-GA levels when treated with Cas13d-S24 or Cas13d-S30, compared with Cas13d-NT30. The DPR knockdown efficiency ranged from approximately 20% to 90% among the different lines (Figure 3G), confirming the effectiveness of the CRISPR/Cas13d system in motor neurons derived from *C9orf72* repeat–associated ALS patients. Moreover, we performed linear regression and correlation analyses to examine the relationship between the Cas13d expression level and the poly-GP/poly-GA knockdown efficiency in the C9-ALS iMN lines. Consistent with the results in the iPSCs, positive correlations were observed between the Cas13d level and the knockdown efficiency for both Cas13d-S24 and Cas13d-S30, as measured by poly-GP and poly-GA levels, in the iMNs (Figure 3, H–J). These results provide compelling evidence that the expression level of the CRISPR/Cas13d system is a critical determinant of its ability to effectively inhibit C9orf72 RAN translation, highlighting the importance of optimizing Cas13d expression levels to maximize the therapeutic potential of this approach for treating C9orf72-ALS/FTD.

Additionally, we conducted RT-qPCR to investigate the potential of the CRISPR/Cas13d system to target and cleave the 3 *C9orf72* transcripts in the C9-ALS iPSC and iMN lines. The results demonstrated that the RNA levels of C9-V1 and C9-V3, which contain the expanded GGGGCC repeat, were significantly decreased by Cas13d-S24 or S30 when compared with NT30 in C9-ALS iPSC line 1 (Supplemental Figure 2B) and C9-ALS iMN line 1 and iMN line 2 (Supplemental Figure 2C). Meanwhile, the RNA level of the C9-V2 transcript, which lacks the GGGGCC repeat, remained unaffected. Interestingly, in the patient iPSC lines 2–4 and iMN lines 3–5, the RNA levels of C9-V1, C9-V2, and C9-V3 transcripts were not significantly affected by the CRISPR/Cas13d system (Supplemental Figure 2, B and C), despite a decrease in poly-GP and poly-GA levels (Figure 3, B and G). Consistently, immunoblotting analysis showed that C9orf72 protein levels did not significantly change across all C9-ALS lines (Supplemental Figure 2, D–G). These data are in accordance with the notion that C9-V2, the predominant transcript accounting for vast majority of C9orf72 protein synthesis (5, 30, 31), is not targeted by the CRISPR/Cas13d system. Notably, iPSC line 2 and iMN line 2 were derived from the same individual, yet the decrease in C9-V1 and C9-V3 was only observed in the iMNs but not in the iPSCs, highlighting the intricate interplay between the CRISPR/Cas13d system and the cellular environment in targeting RNA with higher-order structures. These findings suggest that the knockdown of DPRs by CRISPR/Cas13d may vary depending on the cell type and genetic background, and could involve mechanisms beyond RNA cleavage, including a combination of other mechanisms such as translation repression. Indeed, it has been reported that the secondary structures of crRNA, especially “G”-dependent structures, like G-quadruplex, could diminish the target RNA cleavage by CRISPR/Cas13d (21). Additionally, catalytically deactivated Cas13d has been used to target the ribosomal binding site to inhibit translation (32), and the RNA:RNA base pairing itself could directly cause translation silencing through occlusion of the ribosome binding site (33, 34).

Furthermore, we extended our investigation to normal control iPSC and iMN lines to determine whether the CRISPR/Cas13d system could affect the levels of WT *C9orf72* transcripts and proteins. We cultured 4 iPSC lines derived from 4 normal individuals and differentiated them into motor neurons. These cells were subjected to the same treatments as those applied to the C9-ALS patient iPSC and iMN lines. Consistent with our findings in the HeLa reporter cell lines and HEK293 cells, both *C9orf72* RNA and protein levels remained unaffected in the Cas13d-S24 and Cas13d-S30 groups compared with the non-targeting Cas13d-NT30 group in the 4 normal iPSC and iMN cell lines (Supplemental Figure 2, B–G), indicating a highly selective targeting by the CRISPR/Cas13d system against the expanded alleles in C9-ALS patient cells.

Taken together, our results demonstrate that the CRISPR/Cas13d system effectively and selectively targets the expanded GGGGCC repeat RNA of *C9orf72* and reduces the production of poly-GP and poly-GA resulting from C9orf72 RAN translation in iPSCs and iMNs derived from C9-ALS patients. This is achieved through both RNA cleavage and translational inhibition. Additionally, the knockdown efficiency of the CRISPR/Cas13d system positively correlates with its expression level.

### Multiple factors including repeat lengths influence degradation of GGGGCC repeat RNAs by CRISPR/Cas13d.

**Figure 4:**
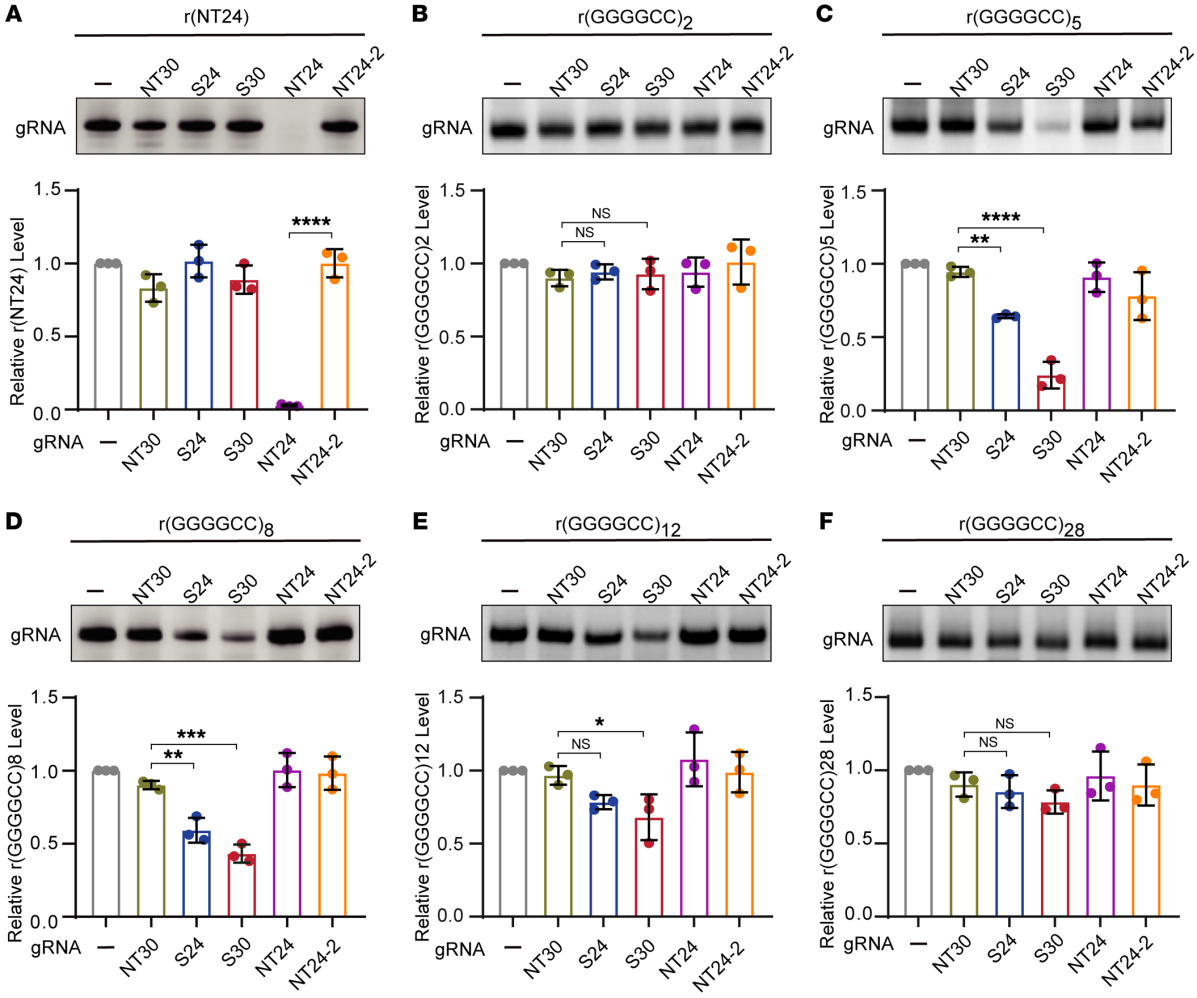
The CRISPR/Cas13d system degraded targetable short but not long GGGGCC repeat RNA. (**A**) Purified Cas13d showed a nearly 100% degradation efficiency of the target RNA r(NT24) with gRNA NT24 but not the other gRNAs, confirming the specificity and high cleavage activity of the Cas13d system. (**B**) The target RNA r(GGGGCC)2 was too short to be degraded by Cas13d. (**C**–**E**) The purified Cas13d showed partial degradation of the target RNAs r(GGGGCC)5 (**C**), r(GGGGCC)8 (**D**), and r(GGGGCC)12 (**E**) with gRNA S24 or S30 but not the other gRNAs, indicating a compromised cleavage activity of Cas13d targeting GGGGCC repeat RNAs and a trend of decreased cleavage efficiency with increased repeat lengths. (**F**) Cas13d was unable to degrade r(GGGGCC)28, demonstrating the limited activity of Cas13d to target or cleave longer GGGGCC repeat RNAs. The cleavage assay was performed in a buffer containing 0.3 μM of gRNA, 0.6 μM of Cas13d protein, and 40 ng/μL of target RNA. Data are presented as means ± SD of 3 independent experiments and were analyzed with unpaired 2-tailed Student’s *t* test (**A**) and ordinary 1-way ANOVA with Dunnett’s multiple-comparison test (**B**–**F**). *P < 0.05, **P < 0.01, ***P < 0.001, ****P < 0.001.

To explore the cleavage activity of Cas13d on GGGGCC repeat RNA in vitro, we expressed Cas13d (molecular weight approximately 110 kDa) in *Escherichia coli*, purified it through nickel–nitrilotriacetic acid (Ni-NTA) affinity chromatography, and analyzed it using SDS-PAGE (Supplemental Figure 3A). Specific and control gRNAs and target RNAs were transcribed in vitro and then incubated with Cas13d protein in a cleavage reaction buffer. We then examined the cleavage activity and specificity of Cas13d using various gRNAs to target the RNA r(NT24) with a random sequence, and we found that only the combination of Cas13d with a perfectly matched gRNA, NT24, showed a nearly 100% degradation efficiency of the target RNA r(NT24) (Figure 4A), while other gRNA combinations, despite occasional mild collateral activities, did not result in such degradation (Figure 4A), confirming the highly specific cleavage activity of the purified Cas13d. Next, we applied the Cas13d system to target GGGGCC repeat RNAs with varied lengths, considering that the length of the gRNA significantly influences Cas13d knockdown efficiency. It has been reported that Cas13d stable targeting binding requires at least 18 nucleotides (nt) of complementarity (35), and 23–30 nt confer the most pronounced target knockdown (21). Indeed, for RNA r(GGGGCC)2, which contains 2× GGGGCC repeat found in most normal individuals (36, 37), the gRNAs S24 and S30 showed no cleavage activity (Figure 4B). Therefore, the 2 units of GGGGCC repeat in normal individuals are unlikely to be targeted by Cas13d, as its 12-nt-length binding site does not provide sufficient length for Cas13d gRNA targeting. This observation aligns with our results in the cells that 14-nt- or 20-nt-length gRNA (S14 and S20) did not demonstrate knockdown efficiency in the EGFP reporter assay (Figure 1C). Consistent with previous observation that gRNAs of 23–30 nt confer the most pronounced target knockdown by Cas13d (21), we found that the RNA r(GGGGCC)5 containing 5× GGGGCC repeat exhibited a robust knockdown of about 80% efficiency by the S30-mediated cleavage (Figure 4C). Notably, with increasing lengths of RNAs, such as r(GGGGCC)8 and r(GGGGCC)12, which contain 8× and 12× GGGGCC repeats, respectively, the gRNAs S24 and S30 exhibited decreasing cleavage activities, ranging from approximately 50% to 20% (Figure 4, D and E). Extending the analysis to a longer GGGGCC repeat RNA, r(GGGGCC)28, carrying 28 repeats, we found that Cas13d was nearly incapable of degrading it (Figure 4F). This observation aligns with previous reports that longer r(GGGGCC)*n* could form an interconnected mesh-like network of aggregated RNAs and RNA condensates through multimolecular G-quadruplexes (38, 39), which have been shown to be resistant to ribonucleases (40).

The Cas13d-mediated degradation of GGGGCC repeat RNAs in the cells appeared to be more complex and may be influenced by multiple factors. By genotyping the repeat sizes of the non-expanded GGGGCC alleles in all the C9-ALS patient and WT control cell lines used in our study through Sanger sequencing, we confirmed that all the WT control cell lines, as well as the HeLa and HEK293 cells, carried 2× GGGGCC repeat, which is the most common form of non-expanded *C9orf72* alleles (Supplemental Table 1). Consistent with the in vitro cleavage assay results (Figure 4B), we did not observe any knockdown of the *C9orf72* transcripts, including the 2× repeat–containing V1 and V3 transcripts, upon the CRISPR/Cas13d treatment (Supplemental Figure 1, C and F, and Supplemental Figure 2, B and C). For the C9-ALS patient cell lines, we observed that the repeat sizes of the non-expanded alleles varied with 2×, 5×, 13×, or 15× GGGGCC (Supplemental Table 1). There were variable changes in the levels of *C9orf72* transcripts in these patient cell lines upon the CRISPR/Cas13d treatment, suggesting potentially complex regulations of both non-expanded alleles and expanded alleles. For example, our RT-qPCR analysis revealed that a subset of C9-ALS patient cell lines, including iPSC line 1 and iMN line 1 and 2, which showed relatively high efficiency in poly-GP or poly-GA knockdown, exhibited a decrease in GGGGCC repeat–containing transcripts (Supplemental Figure 2, B and C), whereas other cell lines with less efficient poly-GP or poly-GA knockdown did not show a significant decrease in these transcripts (Supplemental Figure 2, B and C). Specifically, the C9-ALS iPSC line 1 and iMN line 1 carried a 5× GGGGCC non-expanded allele, making them targetable by S24- and S30-guided Cas13d, as demonstrated by the in vitro cleavage assay (Figure 4C), potentially explaining the reduction of the repeat-containing transcripts C9-V1 and C9-V3 in both cell lines. Intriguingly, the C9-ALS iPSC line 2 and iMN line 2, derived from the same individual with a 2× GGGGCC non-expanded allele, exhibited a mild decrease in C9-V1 and C9-V3 only in the iMNs, suggesting that the expanded allele with hundreds to thousands of repeats can still be partially degraded by the Cas13d system, presumably when local regions of the repeats adopt cleavable conformations under specific cellular environment.

Next, we conducted RNA fluorescence in situ hybridization (RNA FISH) to detect and quantify the RNA foci formed by GGGGCC repeat RNAs in C9-ALS iPSC and iMN lines. The RNA foci were consistently found in all four C9-ALS patient iPSC lines and all five C9-ALS patient iMN lines, but not in the two WT iPSC and iMN lines (Supplemental Figure 3, B and C). Furthermore, quantification of RNA foci in the nuclei of patient cells revealed no significant difference in the treatment with Cas13d-S24 and Cas13d-S30 compared with the non-targeting control treatment with Cas13d-NT30 in each C9-ALS patient cell line (Supplemental Figure 3D), confirming the limited ability of the CRISPR/Cas13d system to degrade the GGGGCC repeat RNAs that are trapped as aggregates within the RNA foci. Collectively, given the significant and consistent knockdown of RAN translation products despite the limited and variable cleavage activities of the CRISPR/Cas13d system against GGGGCC repeat RNAs, these results further support the notion that the suppression of RAN translation by CRISPR/Cas13d involves a combination of RNA targeting and translational repression mechanisms.

### CRISPR/Cas13d suppresses RAN translation in C9orf72 repeat transgenic mice.

**Figure 5:**
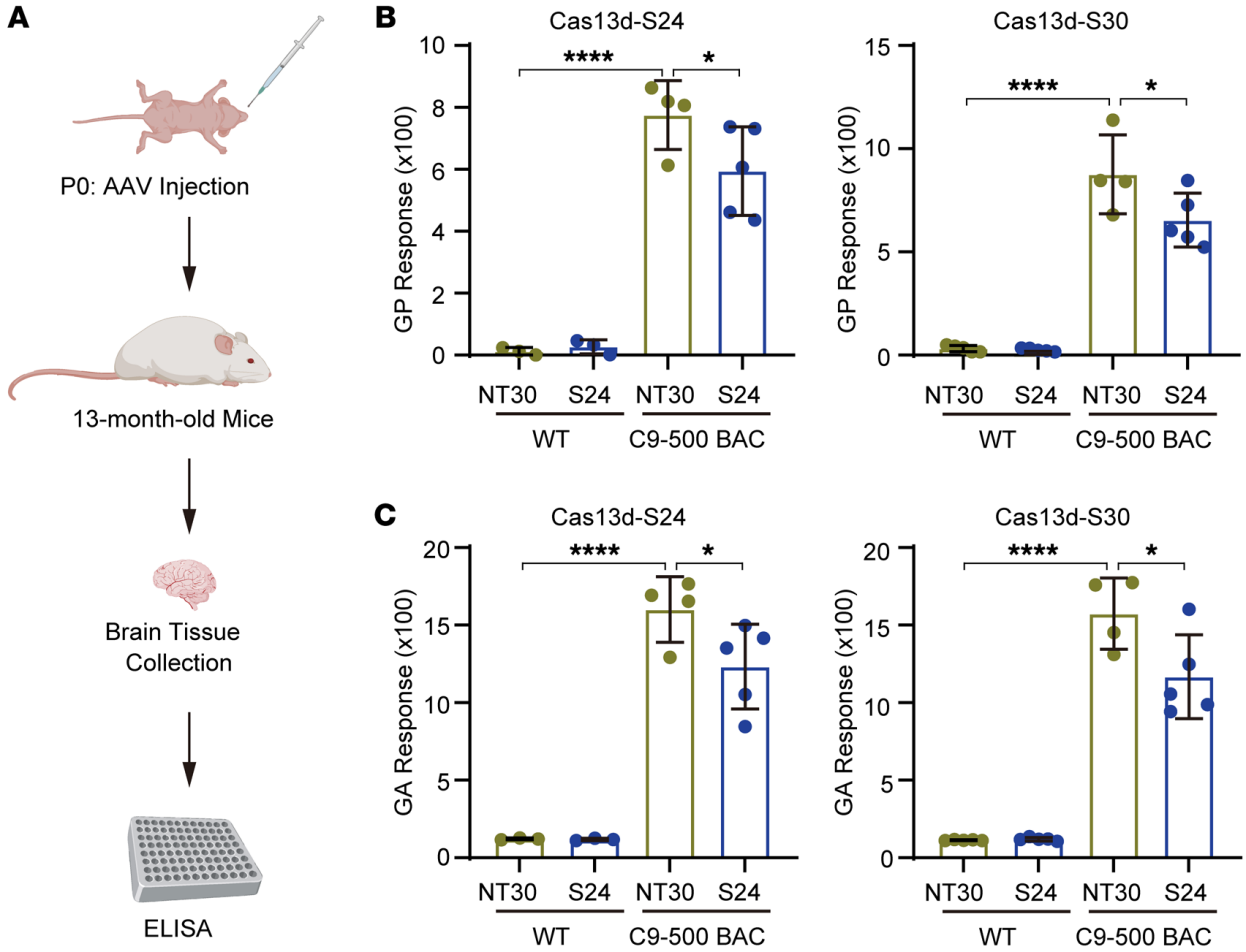
The CRISPR/Cas13d system suppressed RAN translation in C9orf72 repeat transgenic mice. (**A**) Schematic of poly-GP and poly-GA detection in mice treated with AAV9 expressing Cas13d and gRNA. (**B** and **C**) Quantification showed decreased levels of poly-GP (**B**) or poly-GA (**C**) in C9-500 BAC mice but not in the WT mice, when the mice were treated with AAV9 expressing Cas13d-S24 or Cas13d-S30, compared with those treated with control AAV9 expressing the non-targeting Cas13d-NT30. The number of dots in each group indicates the number of mice in the corresponding group. Data are presented as means ± SD and were analyzed with unpaired 1-tailed Student’s *t* test. *P < 0.05, ****P < 0.0001.

To determine whether our approach works in vivo, we used a BAC transgenic mouse model that expresses the human *C9orf72* gene with approximately 500 units of GGGGCC repeat (C9-500) and substantial ﬂanking sequences (41). Although this mouse model has exhibited no survival or motor deficits (42), the mice still express C9orf72 DPRs that can be used as a pathological marker for C9orf72-associated ALS/FTD (4, 42), allowing us to use it to test the efficiency of our CRISPR/Cas13d system in vivo. To deliver the system, we packaged it into adeno-associated virus serotype 9 (AAV9) viral vectors and conducted bilateral intracerebroventricular injection of the Cas13d-NT30, Cas13d-S24, or Cas13d-S30 into the lateral ventricles of C9-500 BAC and WT mice on postnatal day 0 (P0) (Figure 5A). At 13 months of age, the mice were sacrificed, and brain tissues were collected for poly-GP and poly-GA detection (Figure 5A). The C9-500 BAC mice (NT30) exhibited significantly higher poly-GP and poly-GA levels than the WT mice (NT30), confirming the expression of C9orf72 dipeptide proteins in the transgenic mice (Figure 5, B and C). Importantly, the treatment with Cas13d-S24 or Cas13d-S30 led to a significant reduction in poly-GP and poly-GA levels in the C9-500 BAC mice, consistent with the findings observed in C9orf72 repeat–associated ALS patient cells. In addition, we conducted immunohistochemistry (IHC) staining on the mouse brain to detect and quantify the poly-GA inclusion, the most abundantly detected DPR species. The C9-500 BAC mice showed clear poly-GA inclusion signals, while the WT mice had no detectable signals (Supplemental Figure 4A), confirming the specificity of our IHC staining. Moreover, treatment with Cas13d-S24 and Cas13d-S30 resulted in a significant decrease of poly-GA inclusions compared with the non-targeting control, Cas13d-NT30, in the transgenic mice (Supplemental Figure 4A). These results confirm the efficacy of our CRISPR/Cas13d system in both in vitro and in vivo contexts.

Furthermore, we conducted RT-qPCR analysis to quantify the human *C9orf72* transcripts, including C9-V1, C9-V2, and C9-V3, in both WT and C9-500 BAC mice. The results showed that all 3 transcripts were expressed at significantly higher expression levels in C9-500 BAC mice compared with WT mice (Supplemental Figure 4B), confirming the specificity of the primers as well as the mouse genotypes. In our analysis of C9-500 BAC mice treated with different guided Cas13d variants, we found no significant differences in the levels of the three *C9orf72* transcripts when comparing the Cas13d-S24 and Cas13d-S30 groups with the Cas13d-NT30 control group (Supplemental Figure 4B). Additionally, we analyzed the C9orf72 protein level and found no significant differences when comparing Cas13d-S24 and Cas13d-S30 with Cas13d-NT30 (Supplemental Figure 4C). These results are consistent with our observations in the HeLa reporter cell lines, as well as the majority of C9-ALS iPSC and iMN lines studied. Moreover, we performed RNA FISH analysis to detect *C9orf72* GGGGCC repeat RNA foci, and we found no significant differences for the Cas13d-S24 or Cas13d-S30 groups compared with the Cas13d-NT30 control group (Supplemental Figure 4D), in accordance with the results in C9-ALS iPSCs and iMNs (Supplemental Figure 3, B–D).

In summary, the highly consistent results across multiple experimental systems reinforced our conclusion that the CRISPR/Cas13d system showed robust efficiency for selectively targeting GGGGCC repeat RNAs in C9-ALS patient cells and that the knockdown of RAN translation products was achieved through a combination of RNA cleavage and translational repression mechanisms.

## Discussion

RNA-based approaches have long been proposed to be therapeutic strategies for neurodegeneration diseases (43, 44), and the CRISPR/Cas13 system has emerged as a highly effective tool for editing RNA, enabling genetic engineering without modifications to the genomic DNA of organisms. This technology has made an impact in the field of molecular biology and offers potential opportunities for therapeutic applications in various diseases, including neurological disorders (45, 46). Despite the high efficiency of CRISPR/Cas13d in targeting and degrading generic target RNA, it remains a challenge to effectively target RNA molecules adopting higher-order secondary structures (21). A group of diseases known as repeat expansion diseases is characterized by the presence of tandem repeats in the human genome (47), with more than 50 of these disorders being neuromuscular diseases, including ALS, Huntington’s disease, fragile X syndrome, and several spinocerebellar ataxias (47, 48). These repeats, located in either intron or exon regions of the genome, can form intricate higher-order secondary structures in the corresponding RNA transcripts, making them difficult to target using CRISPR/Cas13d.

In this study, we utilized the CRISPR/Cas13 system to specifically target GGGGCC repeat RNAs, which are associated with the development of C9orf72-associated ALS/FTD. We compared the efficiency of two Cas13 variants, Cas13b and Cas13d, and found that Cas13d, with its smaller protein size, exhibited superior knockdown efficiency of GGGGCC repeat RNA when using crRNA S24 or S30. Moreover, we confirmed that the CRISPR/Cas13d system can suppress the translation of C9orf72 RAN proteins by targeting GGGGCC repeat RNA in various cellular models, including C9orf72-associated ALS patient–derived iPSCs and differentiated motor neurons. A strong positive correlation between the Cas13d expression level and the knockdown efficiency of both poly-GP and poly-GA across the C9-ALS iPSC lines provided a clear validation of the specificity of the designed CRISPR/Cas13 system. Lastly, we applied the system in a mouse model expressing the GGGGCC repeats, and we found a significant and consistent reduction of poly-GP and poly-GA levels, confirming the efficiency of our CRISPR/Cas13d system to inhibit C9orf72 RAN translation to produce toxic dipeptide proteins in vivo.

In analyzing the efficiency of the CRISPR/Cas13d treatment, we observed that a subset of C9-ALS patient cell lines, which showed relatively high efficiency in knocking down RAN translation products, exhibited a decrease in repeat-containing transcripts, whereas other cell lines with less efficient poly-dipeptide knockdown did not show a significant decrease in these transcripts. This suggests that both RNA cleavage and translation blocking contribute to the suppression of RAN translation by the Cas13d system. Further analysis revealed that there were multiple mechanisms involved in the regulation of repeat RNA cleavage by the Cas13d system. For example, our in vitro Cas13d cleavage assay and cell-based analyses indicated that 2 units of GGGGCC repeat, which is the most common *C9orf72* allele in the vast majority of normal individuals, are too short to be degraded, thus ensuring the resistance of the normal allele to Cas13d cleavage. Furthermore, CRISPR/Cas13d could partially cleave targetable short-repeat RNAs with 5 to 12 units of GGGGCC repeats but was incapable of degrading longer GGGGCC repeat RNAs, suggesting that repeat length affects the cleavage activity of CRISPR/Cas13d, likely through higher-order structures or assemblies. This finding also raises the potential for the CRISPR/Cas13d system to downregulate C9orf72 transcripts and proteins in a minority of individuals whose WT alleles carry short, but targetable, GGGGCC repeats. Additionally, we examined the formation of RNA foci, which are aggregates formed by GGGGCC repeat RNAs, using RNA fluorescence in situ hybridization. However, we observed no significant changes in the number of foci in cell lines that exhibited a significant reduction in poly-GP and poly-GA as well as GGGGCC repeat–containing transcripts. This demonstrates that GGGGCC repeat RNAs forming aggregates in RNA foci are more resistant to degradation than other GGGGCC repeat RNA–containing transcripts. Moreover, it raises the possibility that GGGGCC repeat RNA may exist in patient cells in a short-repeat form that cannot be detected by RNA fluorescence in situ hybridization but can be degraded by the CRISPR/Cas13d system. Intriguingly, a pair of iPSC and iMN lines, derived from the same individual, exhibited a decrease in repeat RNA–containing transcripts only in the iMN line, but not in the iPSC line, suggesting that repeat RNA cleavage activities of CRISPR/Cas13d could be influenced by cell types. This may be affected by a range of factors such as thousands of RNA-binding proteins, helicases, and abundant post-transcriptional modifications, which have been shown to remodel RNA secondary structures and RNA stability (49, 50), thus highlighting the intricate interplay between the CRISPR/Cas13d system and the cellular environment. These results suggest that the knockdown efficiency of RAN translation products by CRISPR/Cas13d is dependent on genetic background or cellular environment. Importantly, our findings from various cell models, including C9orf72-ALS patient–derived iPSCs, motor neurons, and the repeat expansion mouse model, demonstrate that the reduction of RAN translation products by treatment with the CRISPR/Cas13d system can occur independently of RNA degradation, indicating an underlying mechanism involving translational suppression. Meanwhile, owing to the lack of behavioral phenotypes and other neurodegenerative features in the current C9orf72 animal models, the protective effects of the CRISPR/Cas13d system in C9-ALS/FTD patients require further evaluation.

In summary, our study demonstrates the efficiency of the CRISPR/Cas13d system in targeting GGGGCC repeat RNA to inhibit C9orf72 RAN translation while revealing an underlying mechanism combining both RNA cleavage and translational suppression. The knowledge and insights gained from our studies could be applied to research on other repeat expansion diseases. Furthermore, recent advancements in CRISPR technology have introduced smaller Cas13 variants, such as Cas13X and Cas13bt (51, 52), as well as a promising alternative RNA-targeting system, Cas7-11 (53). These developments expand the RNA-targeting toolbox, and considering our study’s promising and substantial targeting effects on *C9orf72* repeat RNAs using the CRISPR/Cas13d system, it would be worthwhile to explore the application of other CRISPR/Cas systems to target repeat RNAs associated with various diseases in the future.

## Methods

### Sex as a biological variable.

Our study examined male and female animals, and similar findings are reported for both sexes.

### DNA construction.

To construct the Cas13b and Cas13d lentiviral vectors, a BamHI site within the Cas13b coding region from a plasmid (Addgene, 103862) was first disrupted through site-directed mutagenesis using the overlap PCR method. The Cas13b coding sequence from the mutated plasmid and the Cas13d coding sequence from a plasmid (Addgene, 109049) were then individually amplified by PCR and inserted into the lentiviral vector lentiCRISPR v2 (Addgene, 52961) between XbaI and BamHI sites. The crRNA DNA oligonucleotides were synthesized, annealed, and inserted into the lentiviral vector containing the Cas13b coding sequence between BsmBI and EcoRI sites. Similarly, the crRNA DNA was inserted into the lentiviral vector with the Cas13d coding sequence between BsmBI and NheI sites. The reporter construct, comprising 8× GGGGCC repeats followed by the EGFP gene, was generated in the pCDNA3.1 backbone (Thermo Fisher Scientific), as previously described (23). For the C9orf72 RAN translation luciferase-based reporter constructs, the GA-frame, GP-frame, and No-G4C2-repeat control constructs were provided by Shuying Sun (Johns Hopkins University, Baltimore, Maryland, USA) (27). We generated the GR-frame construct by inserting 1 nucleotide shift during PCR amplification of the Nluc gene based on the GA-frame construct at the NotI site. For the construction of AAV-Cas13d vectors, the Cas13d and crRNA DNA sequences, along with their respective promoters from the lentiviral vectors, were amplified and ligated into the AAV vector PX552 (Addgene, 60958) between the MluI and HindIII sites. All the primer crRNA DNA sequences are described in Supplemental Table 2.

### Cell culture and transfection.

Both HEK293 and HeLa Flp-In cells were cultured in DMEM supplemented with 10% (vol/vol) fetal bovine serum at 37°C with 5% CO_2_. For the EGFP reporter assay, HEK293 cells were plated in 12-well plates at 4 × 10^5^ cells per well. The next day, 120 ng EGFP reporter vector and 480 ng lentiviral vector with Cas13b or Cas13d were transfected with 1.2 μL of Lipofectamine 2000 (Invitrogen). Forty-eight hours after the transfection, cells were harvested for immunoblotting analyses and RT-qPCR.

### Dual-luciferase reporter assay.

The dual-luciferase–based C9orf72 RAN translation reporter assay was performed as described previously (23). Brieﬂy, HeLa Flp-In cells were plated in 6-well plates at 8 × 10^5^ cells per well, followed by treatment with 2 μg/mL doxycycline for 24 hours the following day. Then the cells were harvested to measure the Nluc and Fluc luciferase activity by the Nano-Glo Dual-Luciferase Reporter Assay System (Promega) on a BioTek Synergy H1 Hybrid microplate reader. For the RAN translation reporter assay in HEK293, cells were plated in 12-well plates at 4 × 10^5^ cells per well. The next day, 50 ng RAN translation reporter vector and 500 ng lentiviral vector with Cas13d were transfected with 0.55 μL of jetOPTIMUS (Polyplus). Twenty-four hours after the transfection, cells were harvested for luciferase activity analysis as described above, followed by immunoblotting analyses and RT-qPCR.

### Genotyping the non-expanded GGGGCC repeat size.

Genomic DNA was purified with Monarch Genomic DNA Purification Kit (New England Biolabs). A region in the *C9orf72* WT allele containing the non-expanded GGGGCC repeat was amplified by LA Taq DNA polymerase (Takara, RR002A) with the purified genomic DNA as the template. The resulting PCR products were purified and analyzed by Sanger sequencing. Primer sequences are described in Supplemental Table 2.

### RNA fluorescence in situ hybridization.

RNA fluorescence in situ hybridization was performed as previously reported with modifications (23). Briefly, cells grown on coverslips (Deckglaser) were fixed in 3.75% formaldehyde in PBS at room temperature for 10 minutes, then permeabilized in ice-cold 70% ethanol for 30 minutes. For the mouse brain tissues, 10 μm frozen sections were cut on the cryostat and fixed in 3.75% formaldehyde in PBS at room temperature for 20 minutes, followed by permeabilization in ice-cold 70% ethanol for 30 minutes. After a 10-minute rehydration step in wash buffer (40% deionized formamide in 2× SSC), cells were prehybridized at 55°C for 10 minutes in hybridization buffer (40% deionized formamide, 2× SSC, 20 μg/mL BSA, 100 mg/mL dextran sulfate, 10 μg/mL yeast transfer RNA, and 2 mM vanadyl sulfate ribonucleosides). Cells were then incubated at 55°C for 2 hours in hybridization buffer containing 125 nM (CCCCGG)4-Cy3 probe, previously denatured at 95°C for 5 minutes before addition to the buffer. Subsequently, cells were washed 3 times with wash buffer at 55°C for 10 minutes each. Coverslips were then stained with 0.5 μg/mL DAPI (4′,6-diamidino-2-phenylindole) in PBS at room temperature for 10 minutes and mounted with ProLong Gold Antifade (Thermo Fisher Scientific). Images were obtained using a Leica SP8 confocal microscope with 1-μm *z*-step size and matched exposure settings. All solutions were made with diethyl pyrocarbonate–treated water, and formamide was freshly used. The RNA foci were analyzed by ImageJ version 1.53 (NIH).

### Human iPSC and motor neuron cultures.

Human iPSC culturing and motor neuron differentiation were performed as previously described (23). The iPSCs were cultured in StemFlex medium (Thermo Fisher Scientific, A3349401) on plates coated with Matrigel (Corning, 354230). To generate motor neurons, iPSCs were first differentiated into neuroepithelial progenitor (NEP) cells using neural medium (1:1 DMEM/F12: neurobasal medium, GlutaMAX [Thermo Fisher Scientific, 35050061], N2 supplement, B27 supplement, and ascorbic acid) with 3 μM CHIR99021, 2 μM SB431542, and 2 μM DMH-1 for 6 days. NEP cells were then split and cultured in neural medium supplemented with 1 μM CHIR99021, 2 μM SB431542, 2 μM DMH-1, 0.1 μM retinoic acid (RA), and 0.5 μM purmorphamine for an additional 6 days to generate motor neuron progenitor (MNP) cells. MNPs were dissociated using a cell scraper and placed in suspension culture in neural medium supplemented with 0.5 μM RA and 0.1 μM purmorphamine. After an incubation period of 8 days, motor neuron–like cells were dissociated and plated onto Matrigel-coated plates and cultured in neural medium supplemented with 0.5 μM RA, 0.1 μM purmorphamine, and 0.1 μM compound E to attain mature motor neurons. For stably expressing the CRISPR/Cas13d system, iPSCs were transduced with 50 μL of concentrated lentivirus solution per well in 6-well plates. Following a 2-day transduction, cells were cultured in selection medium containing 1 μg/mL puromycin for 7 days before subsequent analysis. For the transduction of iPSC-derived motor neurons (iMNs), lentiviruses were introduced on day 4 of the motor neuron maturation stage and incubated for 2 days, followed by an additional 8 days before harvesting for subsequent analysis. The patient information for the iPSCs is provided in Supplemental Table 1.

### Lentivirus production and transduction.

For lentivirus production, the Cas13d lentiviral vector containing gRNA NT30, S24, or S30 was cotransfected with the packaging plasmid psPAX2 (Addgene, 12260) and the envelope plasmid pMD2.G (Addgene, 12259) into HEK293 cells at a molar ratio of 1:1:1. After transfection, fresh medium was applied 6 hours later, and cells were allowed to proliferate for 48 hours. Subsequently, supernatants containing the virus were collected and filtered through a 0.45 μm membrane (MilliporeSigma, HVHP02500). The filtered supernatants were then combined with 4× lentivirus concentrator solution (1× PBS [pH 7.4] and 40% PEG-8000 [wt/vol]) and left at 4°C overnight. The resultant solution was centrifuged at 1,600*g* for 60 minutes at 4°C, and the virus pellets were resuspended in cold PBS to 1/20 of the original volume, then aliquoted for storage. To establish stable HeLa Flp-In cells expressing the CRISPR/Cas13d system, cells were transduced with 20× concentrated lentiviruses 24 hours after seeding in culture plates. Puromycin (2 μg/mL) was introduced at 48 hours after transduction, and cells were subjected to selection for 7 days before analysis.

### AAV packaging, purification, and transduction.

AAV9 vectors expressing Cas13d and crRNAs were packaged according to a previously described protocol (54). Briefly, 2 helper plasmids, pAdDeltaF6 (Addgene, 112867) and pAAV2/9n (Addgene, 112865), together with Cas13d and crRNA-expressing AAV constructs were transfected into HEK293 cells by jetPRIME (Polyplus-transfection). Six hours later, culturing medium was replaced with fresh medium, and 72 hours later, cells and supernatant medium were collected, followed by chloroform purification. AAV viruses were further concentrated and purified by Amicon Ultra Centrifugal Filters (Millipore, UFC510024), and titers were measured by qPCR using PX552 ITR primer 1 (GGAACCCCTAGTGATGGAGTT) and primer 2 (CGGCCTCAGTGAGCGA).

### In vitro transcription of guide and target RNAs.

For the production of gRNAs, oligonucleotides carrying the T7 promoter and appropriate downstream sequence were synthesized and annealed with an antisense T7 oligonucleotide for crRNAs in annealing buffer (10 mM Tris-HCl, 50 mM LiCl) to be used as T7 transcription templates. For the production of target RNAs including r(NT24), r(GGGGCC)8, and r(GGGGCC)28, the constructs containing the DNA sequences, including non-GQ-48-mer, (GGGGCC)8, and (GGGGCC)28, were described in our previous study (23). Additional constructs for r(GGGGCC)2, r(GGGGCC)5, and r(GGGGCC)12 were generated in the same way as the (GGGGCC)8 construct in our previous study (23). The constructs were linearized by HindIII and purified using the Wizard SV Gel and PCR Clean-Up system (Promega), then used as templates for transcription. The annealed oligonucleotides (40 nM) or 1 μg of linearized plasmids were incubated in transcription buffer (80 mM HEPES-LiOH buﬀer [pH 7.5], 10 mM MgCl_2_, 50 mM LiCl, 10 mM DTT, 1 mM spermidine) supplemented with 4 U/μL T7 RNA polymerase (Thermo Fisher Scientific, EP0111), 2 mM rNTPs, 1 U/μL RiboLock RNase Inhibitor (Thermo Fisher Scientific, EO0381), and 0.025 U/μL pyrophosphatase, inorganic (Thermo Fisher Scientific), for transcription at 37°C for 2 hours. The samples were then treated at 37°C for 30 minutes with DNase I (Thermo Fisher Scientific, EN0521) at a concentration of 0.04 U/μL to remove DNA, followed by addition of EDTA at a final concentration of 20 mM to stop the reaction. RNA transcripts were purified using RNA Clean & Concentrator-25 (Zymo Research) and examined by 10% denaturing urea PAGE.

### Cas13d purification and cleavage reaction.

Cas13d coding sequence was amplified from a plasmid (Addgene, 109049) using a forward primer introducing an NdeI site (TACCACATATGATCGAAAAAAAAAAGTCCTTCGCCAA) and a reverse primer introducing a BamHI site (TTGCAGGATCCTTAGGAATTGCCGGACACCTTCTTTTTCTC). The resulting fragment was ligated into pET28a (Novagen) between the NdeI and BamHI sites. The resulting plasmids were transformed into Rosetta2(DE3) cells (Novagen), induced with 0.1 mM IPTG at OD_600_ 0.4, and grown for 3 hours at 37°C. Cells were harvested and Cas13d protein was purified with Ni-NTA agarose (QIAGEN) under native conditions and quantified with Pierce BCA protein assay (Thermo Fisher Scientific). For the cleavage reaction, 700 ng of purified Cas13d protein was mixed with 60 ng of gRNA at approximately 2:1 molar ratio in 5 μL of Cleavage Buffer (40 mM HEPES [pH 7.2], 1 mM MgCl_2_) and incubated at 37°C for 15 minutes. Then, 400 ng of target RNAs, preheated at 95°C for 5 minutes in 5 μL solution containing 0.1 mM EDTA and cooled on ice, were added. The mixtures were then incubated at 37°C for 1 hour. After addition of 0.5 μL of Proteinase K (New England Biolabs, P8107S) and incubation at 37°C for 15 minutes, the reaction was denatured in loading buffer (80% deionized formamide, 20 mM EDTA) at 95°C for 5 minutes. The mixtures were separated on 15% TBE-urea polyacrylamide gels (19:1 acrylamide/bis) for r(NT)24, r(GGGGCC)2, r(GGGGCC)5, r(GGGGCC)8, and r(GGGGCC)12, or 8% gels for r(GGGGCC)28. Gels were stained with SYBR Gold Nucleic Acid Gel Stain (Thermo Fisher Scientific) before imaging via BioDoc-It Imaging System M-26 (UVP).

### Animals and brain lysate preparation.

The C9-500 transgenic and control WT mice (The Jackson Laboratory, strain 029099) were bred at the Johns Hopkins mouse facilities. Two microliters of AAVs expressing Cas13d and crRNA at a titer of approximately 1.5 × 10^10^ viral genomes per μL were administered by bilateral intracerebroventricular injection into neonatal P0 WT or C9-500 transgenic mice. At 13 months of age, mice were euthanized in a CO_2_ chamber, and brains were harvested by either flash-freezing in liquid nitrogen and then storage at −80°C for RNA and protein analysis, or fixation in 4% paraformaldehyde for IHC. For brain lysates, half brains (excluding cerebellum) of AAV-treated mice were sonicated in ice-cold homogenate buffer (10 mM Tris-HCl [pH 8.0], 1 mM EDTA, 2× Phosphatase Inhibitor Cocktail 2 [MilliporeSigma, P5726], 2× Phosphatase Inhibitor Cocktail 3 [MilliporeSigma, P0044], 2× Protease Inhibitor Cocktail [MilliporeSigma, P8340], 2 mM PMSF) at 1:5 wt/vol ratio with brief pulses at 25% power until tissue was completely dissolved. One hundred microliters of brain homogenate was mixed with 100 μL of 2× lysis buffer (50 mM Tris [pH 7.4], 250 mM NaCl, 2% Triton X-100, 4% SDS, 2× Phosphatase Inhibitor Cocktail 2 [MilliporeSigma, P5726], 2× Phosphatase Inhibitor Cocktail 3 [MilliporeSigma, P0044], 2× Protease Inhibitor Cocktail [MilliporeSigma, P8340], 2 mM PMSF), sonicated at 30-second on/off intervals until the lysate was clear, and spun at 16,000*g* for 20 minutes at 4°C. Supernatants were collected for poly-GP analysis.

### Poly-GP and poly-GA ELISA.

For the poly-GP ELISA, supernatants from cell lysates were collected, and protein concentrations were determined using the Pierce BCA protein assay (Thermo Fisher Scientific). Samples were diluted to a concentration of 1 mg/mL for ELISA following established protocol (23, 28). Briefly, 0.375 μg/mL biotinylated rabbit anti-GP antibody was incubated in 96-well small-spot streptavidin-coated plates at room temperature for 1 hour. After 3 washes with PBST, 35 μL of cell lysate was added per well in duplicates and incubated at room temperature for 3 hours. After another set of PBST washes, sulfo-tagged detection antibodies were introduced at 1 μg/mL and incubated for 1 hour. After further PBST washes, 150 μL of read buffer was added, and the samples were promptly imaged using MESO QuickPlex SQ 120. Specificity was confirmed using lysates from HEK293 cells overexpressing GFP-tagged dipeptide repeat proteins. All reagents used in this procedure were from Meso Scale Discovery.

The poly-GA measurements were performed using the methods described previously (10, 55). Briefly, 0.5 μg/mL of an anti-GA antibody was incubated in 96-well small-spot plates overnight at 4°C. The next day, after 3 washes with PBST, the plate was blocked for 1 hour with 200 μL per well of PBS with casein solution. During blocking, an 8-point calibration curve was prepared by performing of serial dilutions of lysate from HEK293 cells expressing 60× GA in PBS with casein. The individual lysate samples were prepared for testing by dilution of the 1 mg/mL stock lysates 4-fold in PBS with casein. After additional PBST washes, 35 μL of standards and lysate samples were added in duplicates and incubated at room temperature for 2 hours. After another set of PBST washes, 50 μL of detection antibody solution with sulfo-tag–labeled anti-GA detection antibody at 1 μg/mL was added to each well, and the plate was incubated for 1 hour at room temperature. After a final plate wash, 150 μL of Meso Scale Discovery read buffer was added, and the samples were immediately read using MESO QuickPlex SQ 120. The ECL signal level produced is proportional to the amount of poly-GA in the sample, and the concentrations in the lysate samples were interpolated from the calibration curve.

### Reverse transcription and quantitative PCR.

RNA was isolated by RNeasy Plus Mini kit (QIAGEN), followed by reverse transcription using QuantiTect Reverse Transcription Kit (QIAGEN). Quantitative PCR was carried out with PowerUp SYBR Green Master Mix (Thermo Fisher Scientific) using a C1000 Touch thermal cycler with a CFX96 Real-Time System (Bio-Rad). The primer sequences are provided in Supplemental Table 2.

### Western blotting.

Protein samples were resolved on 12% SDS-PAGE gels and transferred to nitrocellulose membranes (Bio-Rad). The membranes underwent blocking in 5% milk in 1× TBST (Tris-buffered saline, 0.1% Tween-20) at room temperature for 1 hour, followed by an incubation with primary antibodies, including anti-GFP (Invitrogen, GF28R; 1:500), anti-C9orf72 (Bio-Rad, VMA00065; 1:1,000), and anti–β-actin (Santa Cruz Biotechnology, sc-47778; 1:1,000), in 5% BSA in 1× TBST containing 0.03% sodium azide at 4°C overnight. After 3 washes with 1× TBST at room temperature for 5 minutes, membranes were incubated with secondary antibodies diluted at 1:1,000 in blocking buffer at room temperature for 2 hours. The secondary antibodies used were goat anti-rabbit IgG IRDye (Licor, IRDye 800CW, 926-32211), donkey anti-mouse IgG (Licor, IRDye 680LT, 926-68022), and anti-mouse HRP-linked IgG (Cell Signaling, 7076S). After another 3 washes with 1× TBST at room temperature for 5 minutes, membranes were imaged on the Odyssey system or GeneGnome XRQ (Syngene), and analyzed using Image Studio version 5.2 (LI-COR).

### Immunostaining.

Matured motor neurons were washed twice with 1× PBS, fixed with 4% formaldehyde at room temperature for 20 minutes, and washed again with 1× PBS. Cells were permeabilized and blocked in a buffer containing 5% normal serum and 0.3% Triton X-100 in 1× PBS at room temperature for 2 hours, and then incubated with anti–choline acetyltransferase (MilliporeSigma, AB144P) at 4°C overnight. The slides were then washed and incubated with a fluorescent secondary antibody at room temperature for 2 hours. After additional PBS washes, the slides were sealed using Prolong Gold Antifade reagent with DAPI (Invitrogen, P36931). Fluorescent images were captured with an SP8 confocal microscope (Leica).

### Immunohistochemistry staining.

Fixed mouse brain tissue was embedded in paraffin and sectioned into slices 5 μm thick. Every fifth section was selected for poly-GA IHC staining. Sections were deparaffinized and hydrated using xylene and graded ethanol. Antigen retrieval was performed using an antigen retrieval buffer (Abcam, catalog ab93684) according to the manufacturer’s instructions. To block nonspecific binding, the slides were incubated with Super Block (ScyTek, AAA125) for 10 minutes, followed by a 1-hour incubation in normal blocking buffer (10% BSA, 0.3% Triton X-100 in PBS). The brain sections were then incubated overnight at 4°C with poly-GA (Proteintech, catalog 24492-I-AP; 1:200) diluted in blocking buffer. After 3 washes with PBS, the slides were incubated in 0.3% H_2_O_2_ in PBS for 15 minutes, then incubated with biotinylated goat anti-rabbit IgG (H + L) (Vector Laboratories, catalog BA-1000; 1:500) diluted in blocking buffer for 1 hour at room temperature. The sections were then incubated with ABC reagent (Vector Laboratories, catalog PK-7100) for 30 minutes at room temperature, followed by signal detection using the DAB kit (Vector Laboratories, catalog K-4100). After counterstaining for nuclei with hematoxylin (MilliporeSigma, catalog 51275), sections were dehydrated in graded ethanol and xylene, mounted in DPX (MilliporeSigma, catalog 06522), sealed with clear nail polish, and imaged with a ×40 objective of a Leica DMi8 fluorescence microscope. Images were manually analyzed using ImageJ (NIH, version 1.53) without intensity or size thresholding.

### Statistics.

Statistical analyses were conducted using GraphPad Prism 8. *P* values less than 0.05 were considered statistically significant. Detailed information on *n* values and other aspects of statistical analysis is provided in the figure legends.

### Study approval.

The mouse protocol was approved by the Animal Care and Use Committee of the Johns Hopkins Medical Institutions.

### Data availability.

All data are included in the article and supplemental material, and values for all data points are provided in the Supporting Data Values file.

## Author contributions

HL performed most of the experiments. XFZ conducted AAV injection, brain tissue collection from mice, and IHC staining. YNL helped with iMN culturing and performed immunoblotting assay. LRH performed and analyzed the poly-GP detection. HL and JW designed the studies and wrote the paper. All authors discussed and helped with the preparation of the manuscript.

## Supplementary Material

Supplemental data

Unedited blot and gel images

Supporting data values

## Version 1 09/17/2024

In-Press Preview

## Version 2 11/01/2024

Electronic publication

## Version 3 12/16/2024

Corrected conflict of interest statement
