# CRISPR/Cas13d targeting suppresses repeat-associated non-AUG translation of C9orf72 hexanucleotide repeat RNA

**DOI:** 10.1172/JCI189439

**Published:** 2024-12-16

**Authors:** Honghe Liu, Xiao-Feng Zhao, Yu-Ning Lu, Lindsey R. Hayes, Jiou Wang

Original citation *J Clin Invest*. 2024;134(21):e179016. https://doi.org/10.1172/JCI179016

Citation for this corrigendum: *J Clin Invest*. 2024;134(24):e189439. https://doi.org/10.1172/JCI189439

During the preparation of this manuscript, an involved patent was mistakenly omitted from the conflict-of-interest statement. The correct statement is below. The HTML and PDF versions of the manuscript have been updated. 

HL and JW are listed as inventors on a patent application (application number: 63313150) filed by Johns Hopkins University on technologies related to this manuscript.

## Version 1 12/16/2024

Electronic publication

