# Corrigendum to CRISPR/Cas13d targeting suppresses repeat-associated non-AUG translation of C9orf72 hexanucleotide repeat RNA

**DOI:** 10.1172/JCI207123

**Published:** 2026-05-01

**Authors:** Honghe Liu, Xiao-Feng Zhao, Yu-Ning Lu, Lindsey R. Hayes, Jiou Wang

Original citation: *J Clin Invest*. 2024;134(21):e179016. https://doi.org/10.1172/JCI179016

Citation for this corrigendum: *J Clin Invest*. 2026;136(9):e207123. https://doi.org/10.1172/JCI207123

After publication of this article, the authors became aware of incorrect figure labels in the supplemental material. In Supplemental Figure 3C, the labels “C9 ALS iPSC Line 2,” “C9 ALS iMN Line 1,” “C9 ALS iPSC Line 2,” and “C9 ALS iPSC Line 2” were incorrect and have been replaced with “C9 ALS iMN Line 2,” “C9 ALS iMN Line 3,” “C9 ALS iMN Line 4,” and “C9 ALS iMN Line 5,” respectively, in the new supplemental material.

The authors regret the error.

## Supplementary Material

Supplemental data

## Version 1 05/01/2026

Electronic publication

