# Supplementary material for: CRISPR/Cas13d targeting suppresses repeat-associated non-AUG translation of C9orf72 hexanucleotide repeat RNA
Source: J Clin Invest. 2024 Sep 17;134(21):e179016. doi: 10.1172/JCI179016 (PMC11527445; doi:10.1172/JCI179016)
Supplement: Supplemental data [file jci-134-179016-s006.pdf]

Supplementary Information for

CRISPR/Cas13d targeting suppresses repeat-associated non-AUG translation of C9orf72 hexanucleotide repeat RNA

Honghe Liu[1,2], Xiao-Feng Zhao[1,2], Yu-Ning Lu[1,2], Lindsey R. Hayes[3], Jiou Wang[1,2]*

1 Department of Biochemistry and Molecular Biology, Bloomberg School of Public Health, Johns Hopkins University, Baltimore, MD 21205, USA

2 Department of Neuroscience, School of Medicine, Johns Hopkins University, Baltimore, MD 21205, USA.

3 Brain Science Institute and Department of Neurology, Johns Hopkins University School of Medicine, Baltimore, MD 21205, USA

This file includes the following information:

Supplemental Figures 1 to 4

Supplemental Tables 1 to 2

Supplemental Figure 1

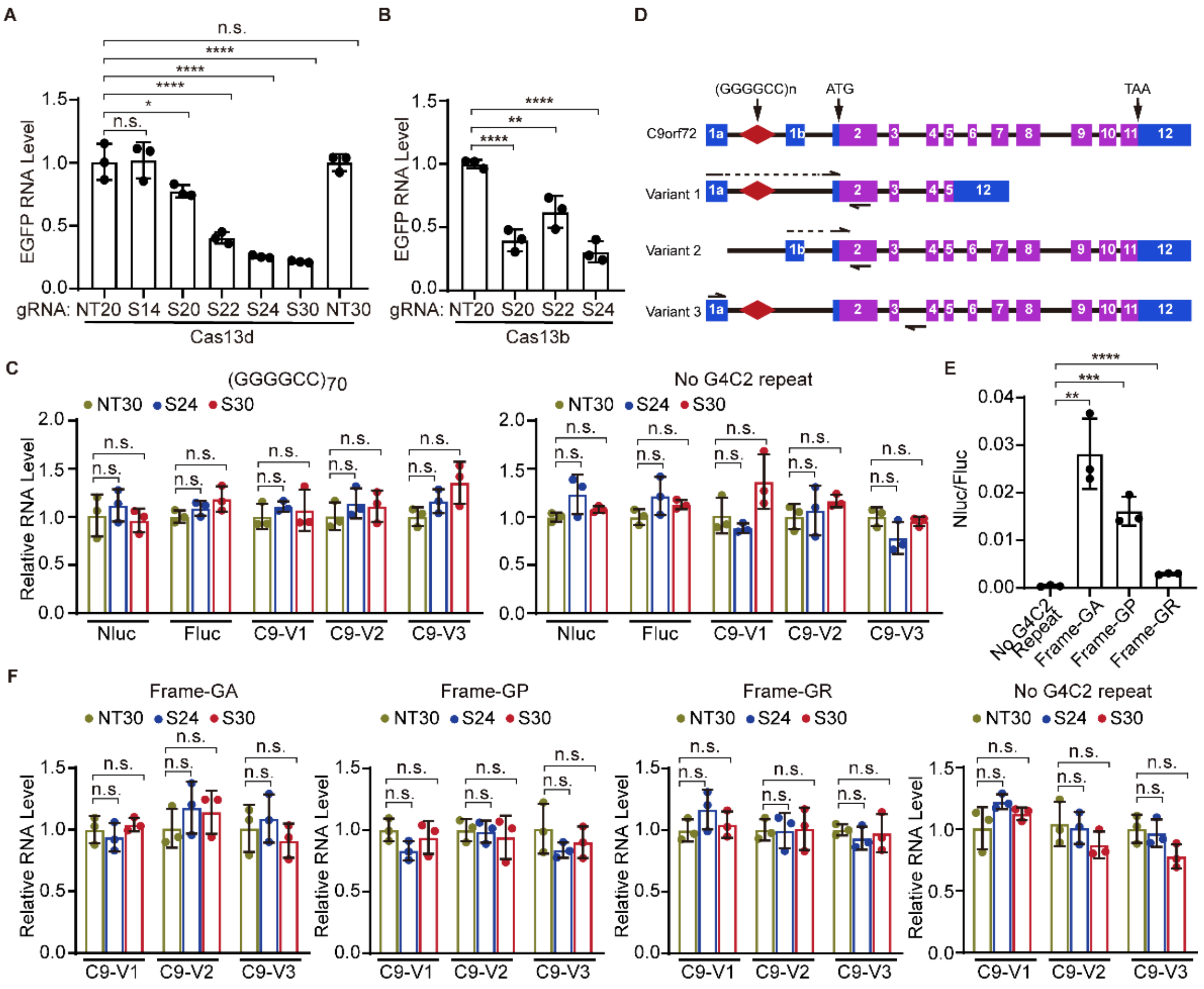

Supplemental Figure 1. Quantification of RNA levels of EGFP, Nluc, Fluc, and three C9orf72 transcripts showed the knockdown of EGFP by specific Cas13 protein or guide RNAs, while the Nluc, Fluc, and three C9orf72 transcripts were not affected by the Cas13 systems in the RAN translation reporter cell lines. EGFP RNA levels were quantified in HEK293 cells transfected with the CRISPR-Cas13d (**A**) or CRISPR-Cas13b (**B**) systems, showing changes consistent with those at the EGFP protein levels presented in Figure 1C. NeoR gene in the CRISPR-Cas13b/d constructs was used as the control gene for qPCR. (**C**) The levels of Nluc, Fluc and three C9orf72 transcripts were not significantly affected in the HeLa

RAN translation reporter cell lines with either (GGGGCC)70 (left) or No-G4C2-repeat (right). (**D**) Schematics of the C9orf72 gene and its three transcript variants. The arrows indicate the locations of primers used for quantifying C9orf72 transcripts. (**E**) Relative RAN translation efficiency (Nano luciferase activity normalized to firefly luciferase) from the GA, GP, or GR frame was significantly higher than the No-G4C2-repeat negative control in HEK293 cells transfected with corresponding constructs. (**F**) The levels of the three C9orf72 transcripts were not significantly changed in the HEK293 cells co-transfected with CRISPR-Cas13d constructs with either the GA-frame, GP-frame, GR-frame or the No-G4C2-repeat control construct. Data are presented as means ± SD of three independent experiments and analyzed with unpaired two-tailed Student's t-test (E) and ordinary one-way ANOVA with Dunnett's multiple comparisons test (A-C and F). **$P < 0.01$, ***$P < 0.001$, ****$P < 0.0001$; "n.s.", no significance.

Supplemental Figure 2

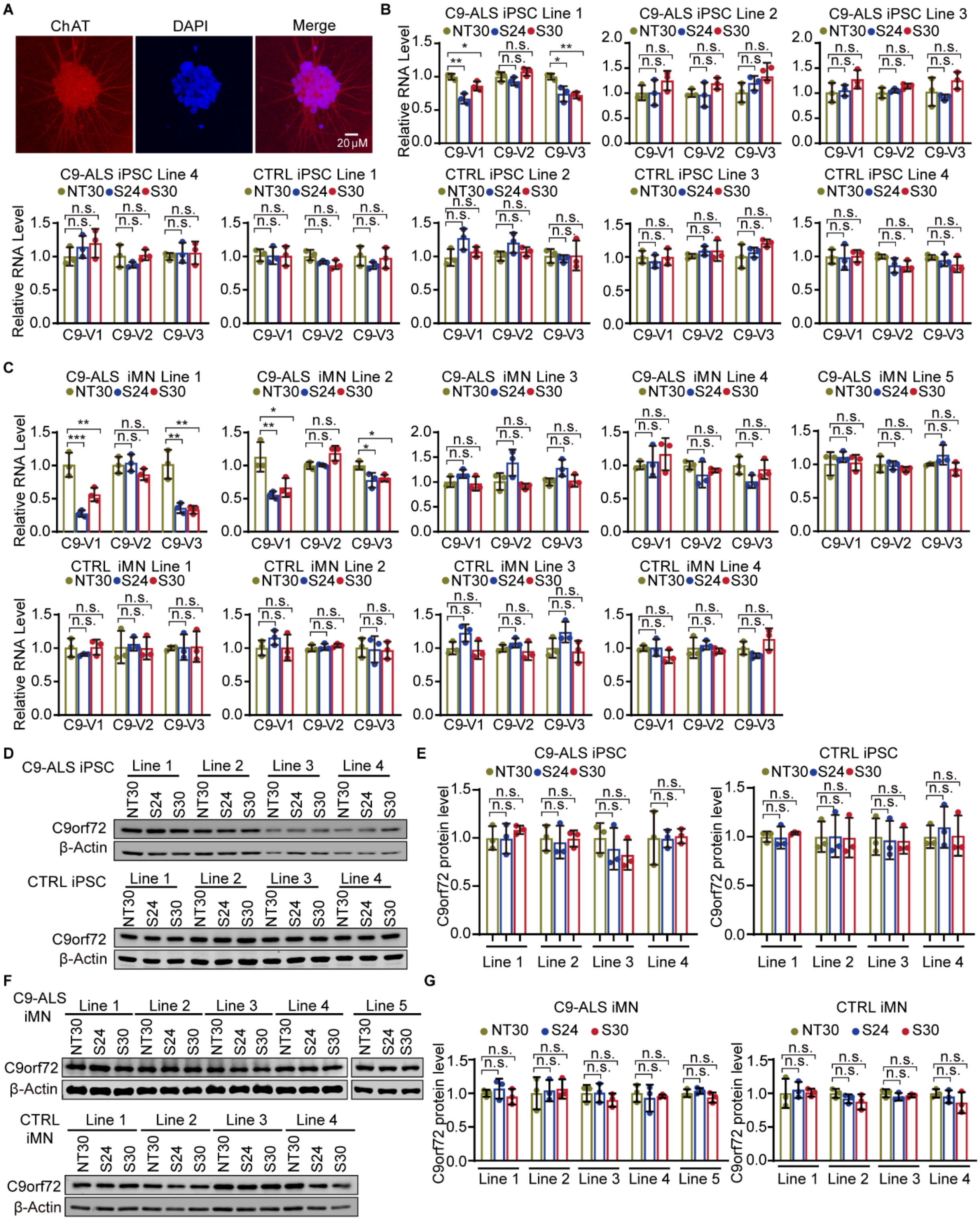

Supplemental Figure 2. Quantification of C9orf72 transcripts in iPSCs and iMNs treated with CRISPR-Cas13d. (**A**) The differentiation of iMNs was confirmed with immunofluorescence staining for the mature motor neuron marker ChAT. (**B**) qPCRs with C9-ALS patient iPSC line 1 stably expressing Cas13d-S24 or Cas13d-S30 showed significant reduction of the C9-V1 and C9-V3 transcripts but not the C9-V2 transcript, while no significant changes were found in the other three C9-ALS patient and the four control iPSC lines. (**C**) qPCRs with C9-ALS iMN line 1 and 2 treated with lentiviruses expressing Cas13d-S24 or Cas13d-S30 showed significant reduction of the C9-V1 and C9-V3 transcripts but not the C9-V2 transcript, while no significant changes were found in the C9-ALS patient iMN lines 3-5 and the control iMN lines 1-4. (**D-E**) Immunoblot analysis of C9orf72 protein showed that the C9orf72 protein level was unaffected in the four C9-ALS patient iPSC and four control iPSC cell lines stably expressing Cas13d-NT30, Cas13d-S24 and Cs13d-S30. (**F-G**) Immunoblot analysis of C9orf72 protein showed that the C9orf72 protein level was unaffected in the four C9-ALS patient iMN and four control iMN cell lines treated with Cas13d-NT30, Cas13d-S24 and Cs13d-S30. Data are presented as means ± SD of three independent experiments and analyzed with ordinary one-way ANOVA with Dunnett's multiple comparisons test. *$P < 0.05$, **$P < 0.01$; "n.s.", no significance.

Supplemental Figure 3

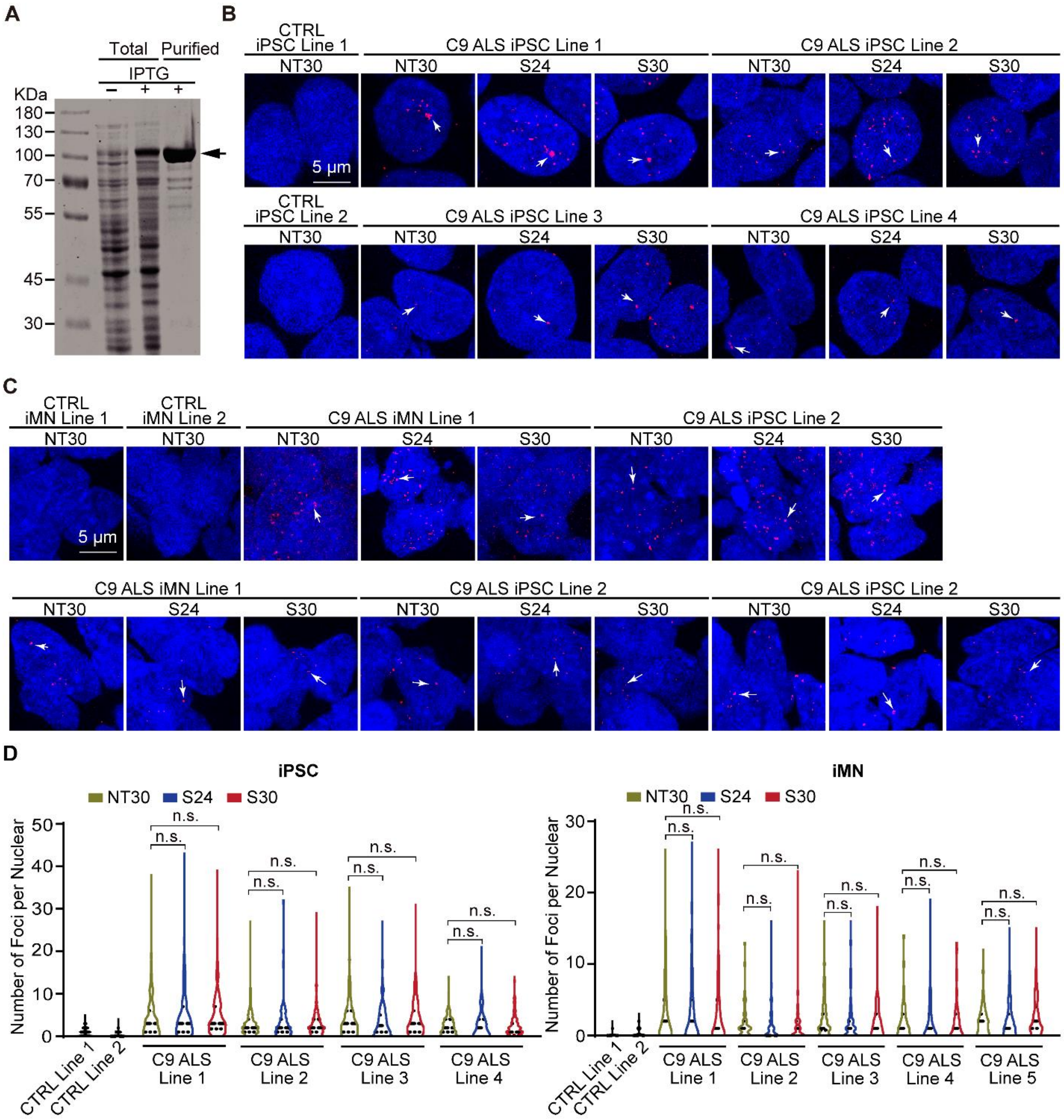

Supplemental Figure 3. CRISPR-Cas13d showed limited ability to degrade RNA foci formed by C9orf72 GGGGCC repeat RNAs. (**A**) SDS-PAGE analysis confirmed the expression and purity of Cas13d protein. Gel was stained by Coomassie blue. (**B**) RNA FISH with a (CCCCGG)4-Cy3 probe demonstrated repeat RNA foci in the C9-ALS patient iPSC cell

lines but not in the control iPSC lines stably expressing guided Cas13d systems. (**C**) RNA FISH with a (CCCCGG)4-Cy3 probe demonstrated repeat RNA foci in the C9-ALS patient iMN lines but not in the control iMN lines treated with lentiviruses expressing guided Cas13d systems. (**D**) Quantification of RNA foci in the nuclei of iPSCs and iMNs treated with CRISPR-Cas13d showed much more foci in ALS-patient iPSCs and iMNs than in control iPSCs and iMNs, respectively, while no significant difference was observed in each cell line when comparing S24- or S30-guided Cas13d with the control NT30-guided Cas13d. For each treatment, at least 160 iPSC cells or 85 iMNs from at least three images were analyzed for the violin plot with ordinary one-way ANOVA with Dunnett's multiple comparisons test. ****$P < 0.0001$; "n.s.", no significance.

Supplemental Figure 4

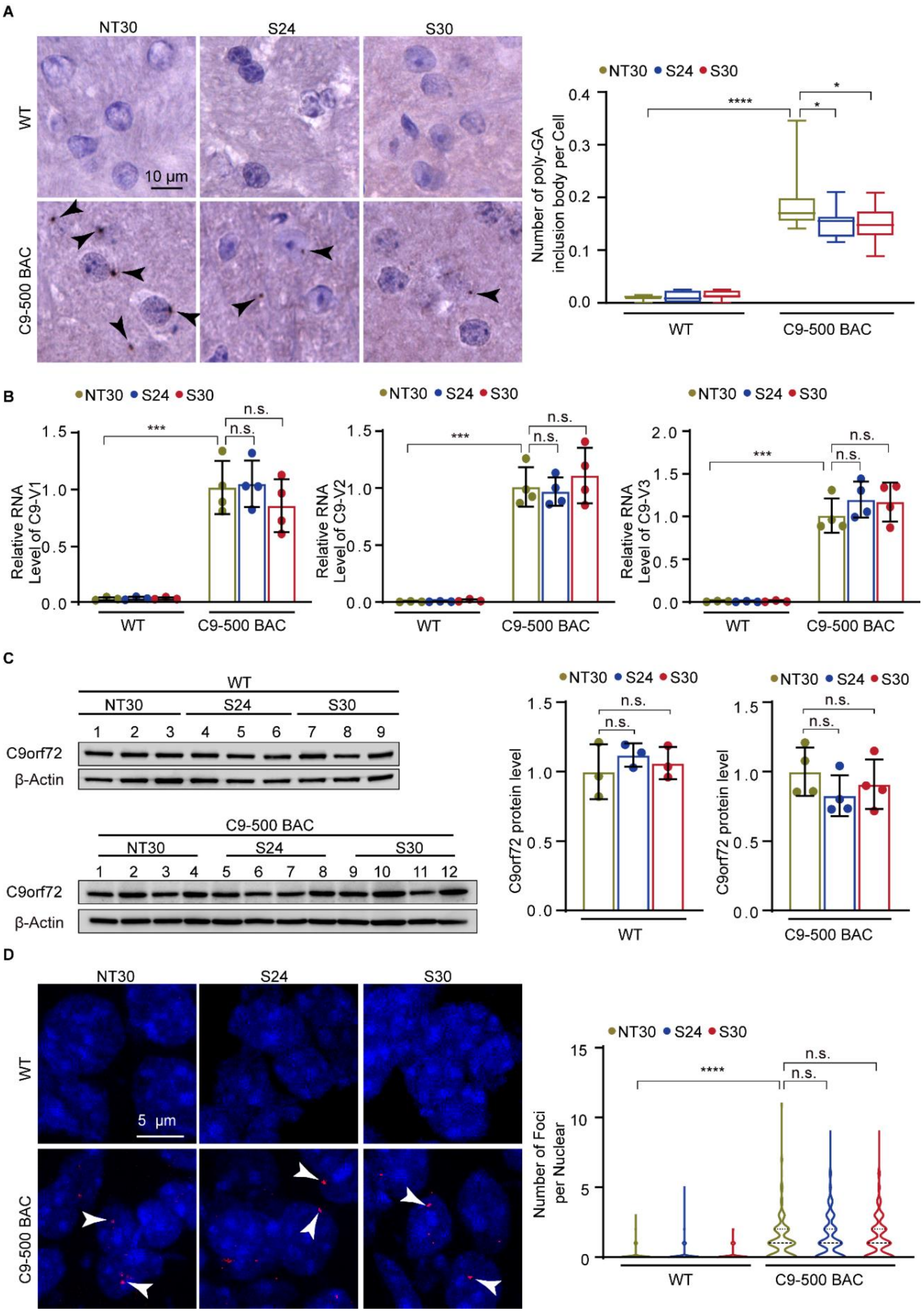

Supplemental Figure 4. Poly-GA inclusions were significantly reduced while C9orf72 transcripts and RNA foci were not significantly changed in the brain tissues of C9-500 BAC mice treated with the AAV-mediated CRISPR-Cas13d system. (**A**) IHC staining showed that poly-GA inclusions were specifically detected in the brain tissues of transgenic C9-500 BAC mice but not the WT mice. The number of poly-GA inclusions in the brains of C9-500 BAC mice treated with Cas13d-S24 and Cas13d-S30 were significantly decreased compared to the non-targeting control treatment Cas13d-NT30. For each group, 6-10 images from two mice were analyzed. Quantitative analysis employed a one-tailed Student's t-test. (**B**) Quantification of C9-V1, C9-V2 and C9-V3 RNA levels showed much higher C9orf72 RNA levels in the transgenic C9-500 BAC mice than the wild-type mice, confirming the primer specificity and mouse genotypes. No significant differences were found in the levels of the three C9orf72 transcripts when comparing Cas13d-S24 or Cas13d-S30 with Cas13d-NT30-treated C9-500 BAC mice. The number of dots in each group indicate the number of mice in the corresponding group. (**C**) Immunoblot analysis of C9orf72 protein showed that the C9orf72 protein level was unaffected in the nine WT and twelve C9-500 BAC mice treated with Cas13d-NT30, Cas13d-S24 and Cs13d-S30. (**D**) RNA foci formed by GGGGCC repeat RNAs were found in the C9-500 BAC mice but not in the wild-type mice, and the number of foci was not significantly changed with the treatment of Cas13d-S24 or Cas13d-S30 compared to the control Cas13d-NT30. For each group, at least 315 cells in three images from three mice were analyzed. Data are presented as box plot with bounds from 25th to 75th percentile, median line, and whiskers ranging from minimum to maximum values and analyzed with unpaired one-tailed t-test (A); means ± SD with unpaired two-tailed t-test (B); or ordinary one-way ANOVA with Dunnett's multiple comparisons test (C); or violin plot with unpaired two-tailed t-test (D). *P < 0.05, ***P < 0.001, ****P < 0.0001; "n.s.", no significance.

Supplemental Table 1. Cell lines used in this study

iPSCs and iMNs							
Cell Line ID	Subject ID	Genotype	Clinical Status	Gender	Age	Source	Repeat size in WT allele
C9-ALS Line 1	ND10689	C9orf72	Affected	F	51	NINDS	5
C9-ALS Line 2	ND12099	C9orf72	Affected	M	49	NINDS	2
C9-ALS Line 3	NDS00239	C9orf72	Affected	F	64	NINDS	2
C9-ALS Line 4	NDS00247	C9orf72	At risk	M	60	NINDS	15
C9-ALS Line 5	ND06769	C9orf72	Affected	F	46	NINDS	13
CTRL Line 1	ND41865	WT	Control	M	64	NINDS	2
CTRL Line 2	GM25256	WT	Control	M	30	Coriell	2
CTRL Line 3	NDS00241	WT	Control	M	36	NINDS	2
CTRL Line 4	NDS00242	WT	Control	F	49	NINDS	2

Other Cell Lines		
Cell Lines	Source	Repeat size in WT allele
Hela	ATCC	2
HEK 293	ATCC	2

Abbreviations: F (Female), M (Male), NINDS (National Institute of Neurological Disease and Stroke), WT (Wild-Type), ATCC (American Type Culture Collection).

Supplemental Table 2. DNA and RNA sequence

Cas13b mutation	Cas13b-mut-F	GCTGCCCAAGGACCGTATCCACAGCGAGAAG
	Cas13b-mut-R	CTTCTCGCTGTGGATACGGTCCTTGGGCAGCT
	Cas13b-F	GAACTCTAGAGCCACCATGGCTCCCAAAAAGAAGAGGAAAGTGGGTTCCATGAACATCCCCGCTCTGGTG
	Cas13b-R	TACATGGATCCCTTCATGATGGCGTACTCC
Cas13b amplification for lentiviral vector	Forward Primer	GAACTCTAGAGCCACCATGGCTCCCAAAAAGAAGAGGAAAGTGGGTTCCATGAACATCCCCGCTCTGGTG
	Reverse Primer	TATCTGGATCCTACCTTCCTCTTCTTCTTGGGAGAGCCCTTCATGATGGCGTACTCC
Cas13d amplification for lentiviral vector	Forward Primer	TCATATCTAGAGCCACCATGAGCCCCAAGAAGAAG
	Reverse Primer	TTGACGGATCCCACCTTCCTCTTTTTCTTAGGTCCAGATCCGGAATTGCC
crRNA	Case3b_NT20	GUGAUAAGUGGAAUGCCAUGGUUGUGGAAGGUCCAGUUUUGAGGGGCUAUUACAAC
	Case3b_S20	CCCCGGCCCCGGCCCCGGCCGUUGUGGAAGGUCCAGUUUUGAGGGGCUAUUACAAC
	Case3b_S22	CCCGGCCCCGGCCCCGGCCCCGUUGUGGAAGGUCCAGUUUUGAGGGGCUAUUACAAC
	Case3b_S24	GGCCCCGGCCCCGGCCCCGGCCCCGUUGUGGAAGGUCCAGUUUUGAGGGGCUAUUACAAC

	Cas13d_NT20	AACCCCUACCAACUGGUCGGGGUUUGAAACGUGAUAAGUGGAAUGCCAUG
	Cas13d_S14	AACCCCUACCAACUGGUCGGGGUUUGAAACCCCCGGCCCCGGCC
	Cas13d_S20	AACCCCUACCAACUGGUCGGGGUUUGAAACCCCCGGCCCCGGCCCCGGCC
	Cas13d_S22	AACCCCUACCAACUGGUCGGGGUUUGAAACCCCCGGCCCCGGCCCCGGCCCC
	Cas13d_S24	AACCCCUACCAACUGGUCGGGGUUUGAAACCCGGCCCCGGCCCCGGCCCCGGCC
	Cas13d_S30	AACCCCUACCAACUGGUCGGGGUUUGAAACCCGGCCCCGGCCCCGGCCCCGGCCCCGGCC
GR-frame construct	GA-F	TAGATTCGCGGCCGCGTCTTCACACTCGAAGATTTCG
	GA-R	GGTATCTTGTCGTCGTCGTCCTTGT
Repeat size PCR	Forward Primer	GCCCACGTAAAAGATGACGC
	Reverse Primer	AGTCGCTAGAGGCGAAAGC
Cas13d amplification for AAV vector construction	MluI-U6-F	ACTAGACGCGTGAGGGCCTATTTCCCATGATTC
	HindIII-Cas13d-R	ATGAGAAGCTTTTACACCTTCCTCTTTTTCTTAGGTCCAG
gRNA DNA oligonucleotides	T7-NT30-F	GACCTCTAATACGACTCACTATAGGAACCCCTACCAACTGGTCGGGGTTTGAAACTCACCAGAAGCGTACCATACTCACGAACAG
	T7-NT30-R	CTGTTCGTGAGTATGGTACGCTTCTGGTGAGTTTCAAACCCCGACCAGTTGGTAGGGGTTCCTATAGTGAGTCGTATTAGAGGTC
	T7-S24-F	GACCTCTAATACGACTCACTATAGGAACCCCTACCAACTGGTCGGGGTTTGAAACCCGGCCCCGGCCCCGGCCCCGGCC
	T7-S24-R	GGCCGGGGCCGGGGCCGGGGCCGGGTTTCAAACCCCGACCAGTTGGTAGGGGTTCCTATAGTGAGTCGTATTAGAGGTC
	T7-S30-F	GACCTCTAATACGACTCACTATAGGAACCCCTACCAACTGGTCGGGGTTTGAAACCCGGCCCCGGCCCCGGCCCCGGCCCCGGCC
	T7-S30-R	GGCCGGGGCCGGGGCCGGGGCCGGGGCCGGGTTTCAAACCCCGACCAGTTGGTAGGGGTTCCTATAGTGAGTCGTATTAGAGGTC
	T7-S24-M-F	GACCTCTAATACGACTCACTATAGGAACCCCTACCAACTGGTCGGGGTTTGAAACGGACACGGACACGGACACGGACAC
	T7-S24-M-R	GTGTCCGTGTCCGTGTCCGTGTCCGTTTCAAACCCCGACCAGTTGGTAGGGGTTCCTATAGTGAGTCGTATTAGAGGTC
	T7-S24-M2-F	GACCTCTAATACGACTCACTATAGGAACCCCTACCAACTGGTCGGGGTTTGAAACTGGCATCTTCAGAATTCTCTACCA
	T7-S24-M2-R	TGGTAGAGAATTCTGAAGATGCCAGTTTCAAACCCCGACCAGTTGGTAGGGGTTCCTATAGTGAGTCGTATTAGAGGTC
qPCR primers	EGFP-qF	GACGTAAACGGCCACAAGTT
	EGFP-qR	AAGTCGTGCTGCTTCATGTG
	NeoR-qF	ACCTTGCTCCTGCCGAGAAAGTAT

	NeoR-qR	ATGTTTCGCTTGGTGGTCGAATGG
	C9_V1_qF	CCACGTAAAAGATGACGCTTGATA
	C9_V2_qF	CGGTGGCGAGTGGATATCTC
	C9_V3_qF	GCAAGAGCAGGTGTGGGTTT
	C9_V123_qR	TGGGCAAAGAGTCGACATCA
	GAPDH-qF	GTCTCCTCTGACTTCAACAGCG
	GAPDH-qR	ACCACCCTGTTGCTGTAGCCAA
	Cas13d-qF	GATGTTTCCGCCTTCAGCA
	Cas13d-qR	CCACGAACTTAGCGTTGACT
	Ms_GAPDH-qF	CATCACTGCCACCCAGAAGACTG
	Ms_GAPDH-qR	ATGCCAGTGAGCTTCCCGTTCAG
