# Supplementary material for: CRISPR/Cas13d targeting suppresses repeat-associated non-AUG translation of C9orf72 hexanucleotide repeat RNA
Source: J Clin Invest. 2024 Sep 17;134(21):e179016. doi: 10.1172/JCI179016 (PMC11527445; doi:10.1172/JCI179016)

Figure 1C, 1D

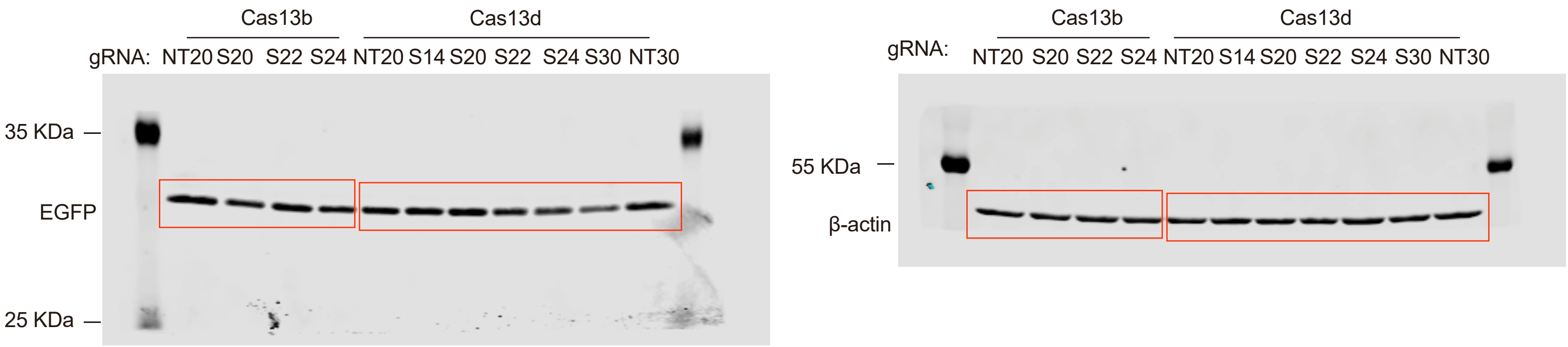

Figure 2D

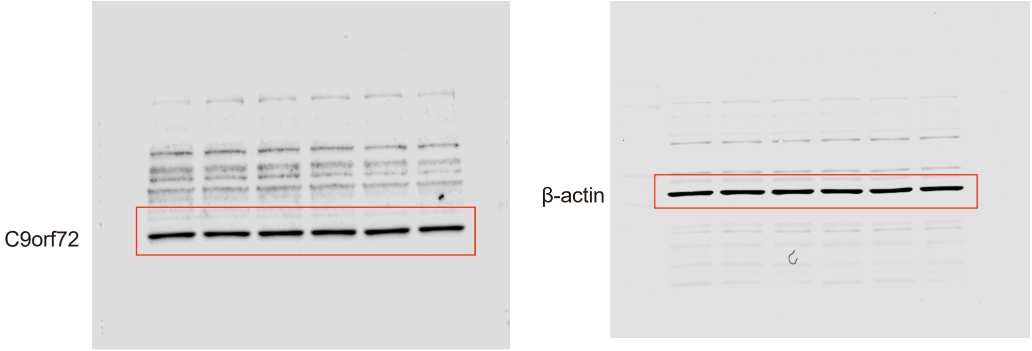

Figure 2F

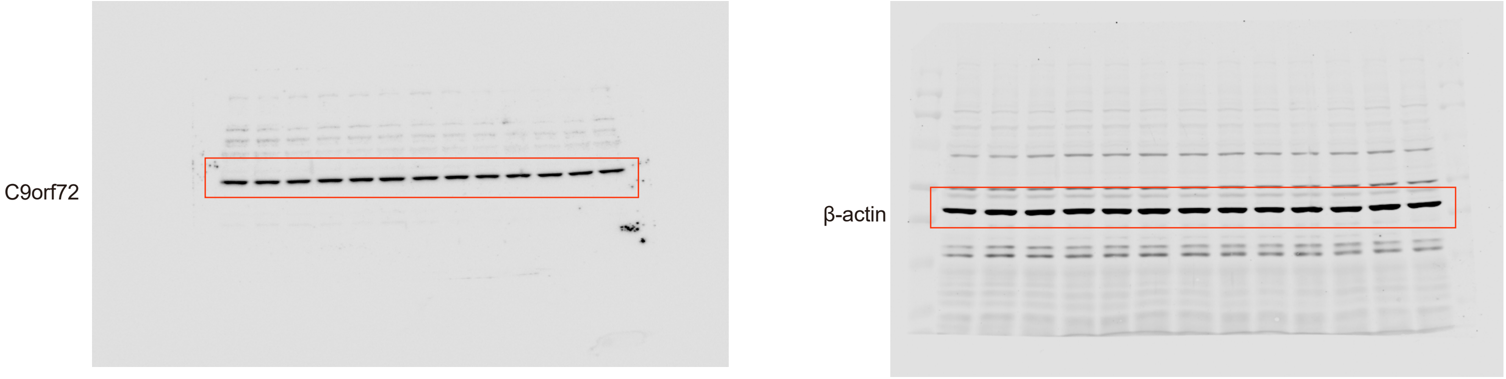

Figure 4A **Figure 4D**

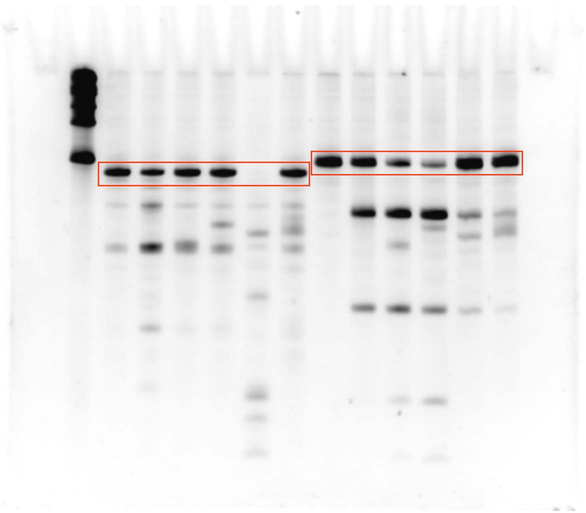

Figure 4B **Figure 4C**

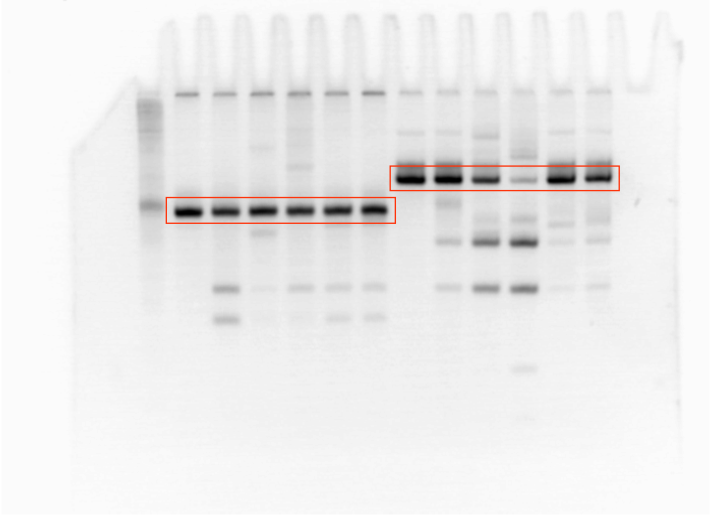

Figure 4E

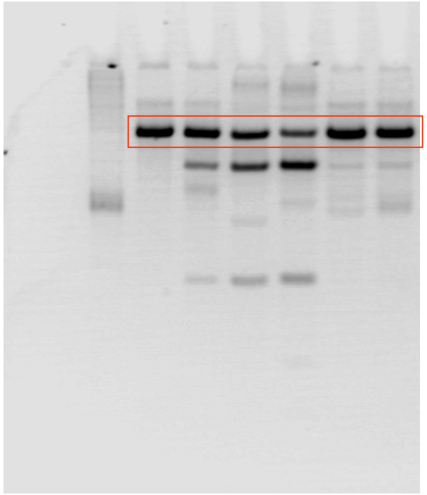

Figure 4F

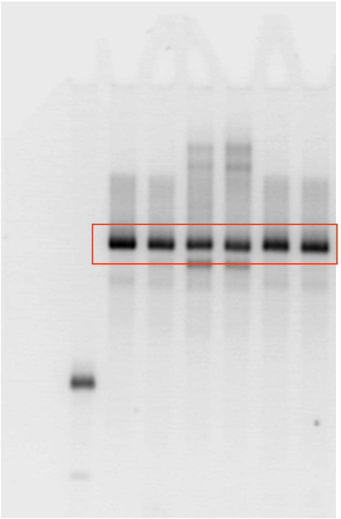

Figure S2C

C9-ALS iPSC

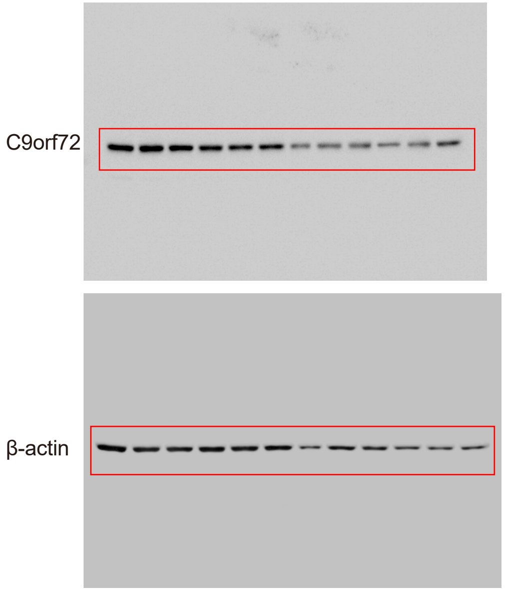

CTRL iPSC

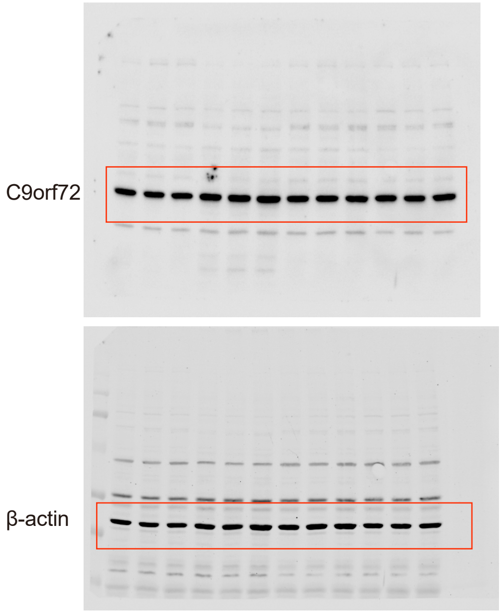

Figure S2F

CTRL iMN

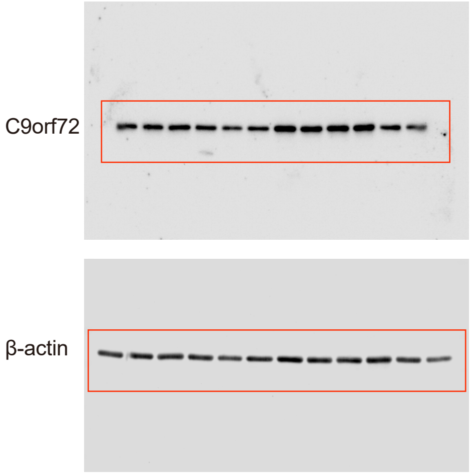

C9-ALS iMN

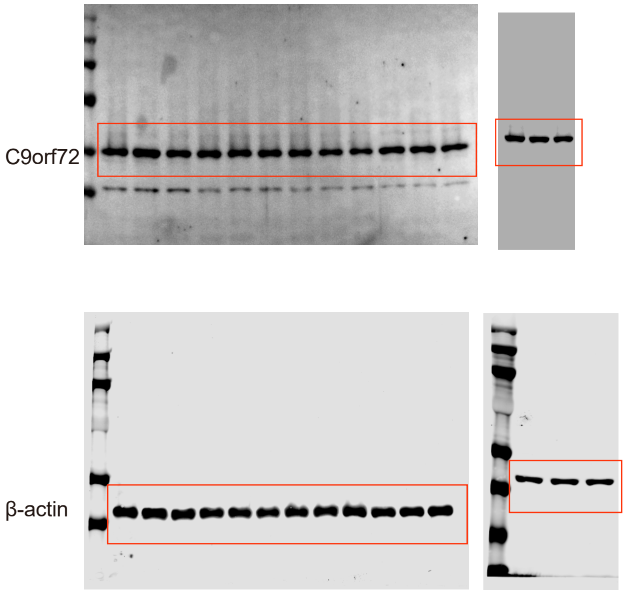

Figure S3A

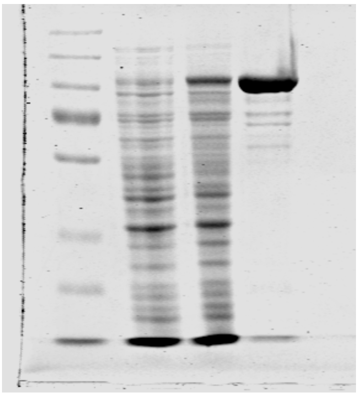

Figure S4C

WT

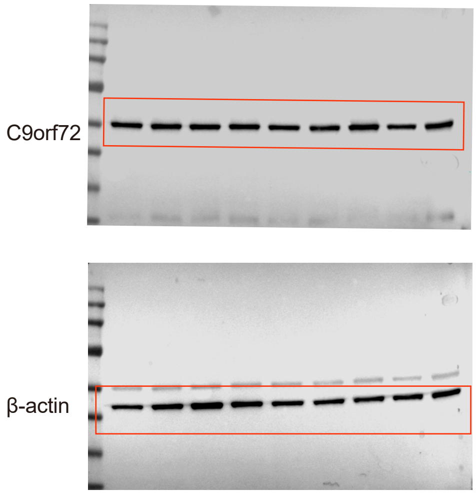

C9-500 BAC

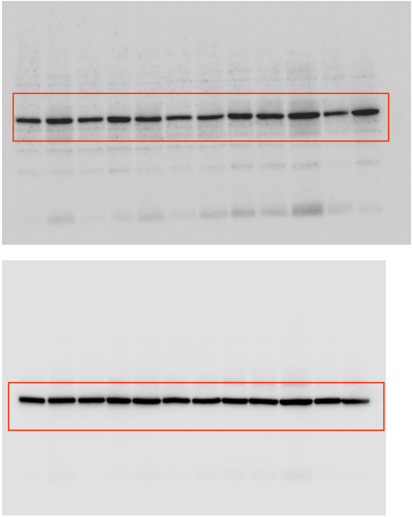

Supplement: Unedited blot and gel images [file jci-134-179016-s007.pdf]
